# Hydroxamic Acids Containing a Bicyclic Pinane Backbone as Epigenetic and Metabolic Regulators: Synergizing Agents to Overcome Cisplatin Resistance

**DOI:** 10.3390/cancers15204985

**Published:** 2023-10-14

**Authors:** Yulia Aleksandrova, Aldar Munkuev, Evgenii Mozhaitsev, Evgeniy Suslov, Konstantin Volcho, Nariman Salakhutdinov, Margarita Neganova

**Affiliations:** 1 Institute of Physiologically Active Compounds at Federal Research Center of Problems of Chemical Physics and Medicinal Chemistry, Russian Academy of Sciences, Severnij Pr. 1, 142432 Chernogolovka, Russia; aleksandrova@ipac.ac.ru; 2 Department of Medicinal Chemistry, N. N. Vorozhtsov Novosibirsk Institute of Organic Chemistry, Siberian Branch, Russian Academy of Sciences, Lavrentiev Ave., 9, 630090 Novosibirsk, Russia; amunkuev@nioch.nsc.ru (A.M.); mozh@nioch.nsc.ru (E.M.); suslov@nioch.nsc.ru (E.S.); volcho@nioch.nsc.ru (K.V.); anvar@nioch.nsc.ru (N.S.)

**Keywords:** hydroxamic acids, cancer, multidrug resistance, epigenetic alterations, metabolism reprogramming

## Abstract

**Simple Summary:**

Chemotherapy is one of the most widely used cancer treatments. A significant barrier to its successful use is the high risk of acquiring the phenomenon of multidrug resistance in cancer. In this regard, today, researchers’ attention is focused on solving this critically important problem. In our work, the approach of using hydroxamic acids containing a para-substituted cinnamic acid core and bearing bicyclic pinane scaffolds, including derivatives of (−)-myrtenol, (+)-myrtenol and (−)-nopol, as a Cap-group is considered to be one of the possible solutions to this problem. Being modulators of epigenetic function and the metabolic state of neoplastic cells, **18c** synergizes with cisplatin to increase the anticancer effect of cytostatic agent, and it overcomes cisplatin.

**Abstract:**

Multidrug resistance is the dominant obstacle to effective chemotherapy for malignant neoplasms. It is well known that neoplastic cells use a wide range of adaptive mechanisms to form and maintain resistance against antitumor agents, which makes it urgent to identify promising therapies to solve this problem. Hydroxamic acids are biologically active compounds and in recent years have been actively considered to be potentially promising drugs of various pharmacological applications. In this paper, we synthesized a number of hydroxamic acids containing a p-substituted cinnamic acid core and bearing bicyclic pinane fragments, including derivatives of (−)-myrtenol, (+)-myrtenol and (−)-nopol, as a Cap-group. Among the synthesized compounds, the most promising hydroxamic acid was identified, containing a fragment of (−)-nopol in the Cap group **18c**. This compound synergizes with cisplatin to increase its anticancer effect and overcomes cisplatin resistance, which may be associated with the inhibition of histone deacetylase 1 and glycolytic function. Taken together, our results demonstrate that the use of hydroxamic acids with a bicyclic pinane backbone can be considered to be an effective approach to the eradication of tumor cells and overcoming drug resistance in the treatment of malignant neoplasms.

## 1. Introduction

Oncological diseases are the cause of approximately 20% of deaths worldwide, occupying a leading position in the register of morbidity and mortality factors [1,2,3]. Despite the fact that there are a huge number of variations of therapeutic strategies, chemotherapy is still the most common and preferred treatment method [4]. A critical limitation in its use is multidrug resistance [5,6], which often occurs after prolonged use of chemotherapeutic agents and is expressed by a decrease in the sensitivity of tumor cells to the action of drugs, leading to relapse and the rapid death of patients [7]. In this regard, intensive efforts are currently being made to develop medicinal agents that could reverse therapeutic resistance and increase the effectiveness of treatment without increasing side effects on a healthy microenvironment [8,9,10,11,12].

**Figure 1:**
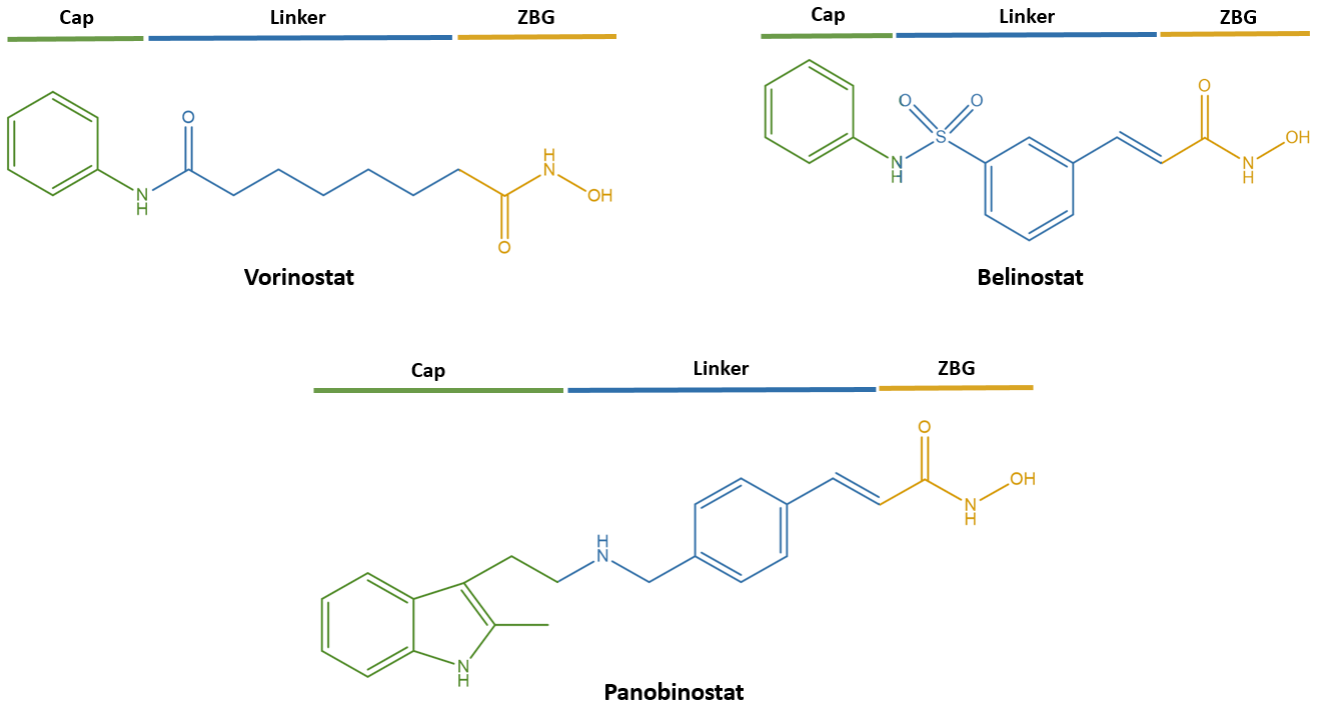
FDA-approved histone deacetylase inhibitors (HDACi) for the treatment of malignant neoplasms.

To date, compounds based on hydroxamic acids with a promising profile of pharmacological activity [13] are actively considered to be valuable agents for the treatment of malignant neoplasms [14]. So, currently, in clinical practice, three representatives of this class are already actively used as powerful antitumor agents due to their excellent inhibitory properties against histone deacetylases (HDACs) for the treatment of peripheral T-cell lymphoma and multiple myeloma: vorinostat (suberoylanilide hydroxamic acid, SAHA) [15], belinostat (PXD101) [16] and panobinostat (LBH 589) [17] (Figure 1).

These compounds correspond to the classical pharmacophore model of HDACs inhibitors, consisting of the following fragments (Figure 1): (1) a zinc-binding group (ZBG) responsible for coordinating Zn^2+^ in the active center of the enzyme, (2) a Cap group interacting with the surface amino acids of the HDACs binding site and (3) a linker necessary for ZBG binding with the Cap group [18].

In recent years, the strategy of combining two or more pharmacophore fragments of various therapeutic applications in one molecule has been gaining momentum in the creation of innovative drugs [19,20,21,22,23,24,25,26]. Such hybrid molecules can simultaneously affect a number of targets involved in the pathogenesis of malignant neoplasms. Thus, the approach in our work of the modification of hydroxamic acids by monoterpene fragments suggests the unconditional contribution of the latter to the expansion of antitumor properties [27,28]. The biological activity of members of this class is widely considered in the literature, where the multifaceted action mechanisms in the chemoprophylaxis of oncological diseases is described. In particular, the success of the clinical use of carvone—a cyclic saturated monoterpenoid—has been confirmed in the treatment of colorectal carcinoma [29], breast adenocarcinoma [30,31], stomach cancer [32], melanoma [33], neuroblastoma [34] and leukemia [35]. An improvement in the pathophysiological state was shown in rats with colon cancer [36] and bladder cancer [37] in the case of the use of a bicyclic monoterpenoid myrtenal. And for another representative of monoterpenes, limonene [38], contained in citrus essential oils, the ability to eliminate multidrug resistance is well known [39], which confirms the prospects of the synthetic approach we used.

Thus, in this paper, we report on the development, synthesis, and evaluation of the biological properties of new hydroxamic acids synthesized as promising therapeutic agents of antitumor orientation.

## 2. Materials and Methods

### 2.1. Chemistry

All reactions were performed with commercially available reagents ((Sigma Aldrich (St. Louis, MO, USA), Acros Organics (Geel, Belgium)). Bruker AV-300 spectrometer (Bruker Corporation, Billerica, MA, USA) (300.13 MHz and 75.46 MHz, respectively), Bruker AV-400 (Bruker Corporation, Billerica, MA, USA) (400.13 MHz and 100.61 MHz), Bruker DRX-500 (Bruker Corporation, Billerica, MA, USA) (500.13 MHz and 125.76 MHz) and Bruker Avance—III 600 (Bruker Corporation, Billerica, MA, USA) (600.30 MHz and 150.95 MHz) were used to record ^1^H and ^13^C NMR spectra. Mass spectra (70 eV) were recorded on a DFS Thermo Scientific high-resolution mass spectrometer. Optical rotations were measured with A PolAAr 3005 polarimeter (Optical Activity, Ramsey, UK). A Mettler Toledo FP900 Thermosystem apparatus was used to measure melting points. Merck silica gel (Merck, Darmstadt, Germany, 63–200 µm) was used for column chromatography. Spectral and analytical measurements were carried out at the Multi-Access Chemical Service Center of Siberian Branch of Russian Academy of Sciences (SB RAS).

Synthesis of (+)-myrtenol **7**

Oxidation of (+)-α-pinene by SeO_2_ in the presence of *t*-BuOOH was carried out as described in [40]. The crude product was treated with NaBH_4_ in MeOH as described in [41]. (+)-Myrtenol was isolated by column chromatography on silica gel (hexane/EtOAc) with 48% overall yield. Spectroscopic data were consistent with those previously reported [41].

Synthesis of Bromides **2** and **8**

To a solution of myrtenol (4.0 g, 26.3 mmol) in Et_2_O cooled to 0 °C, 1.0 mL of PBr_3_ (1.0 mL, 10.5 mmol) was added. The reaction mixture was stirred at 0 °C for approximately 4 h; then, water was added. The organic phase was separated and washed with brine and dried over Na_2_SO_4_. The products isolated in a slightly yellow oil were used in the next step without purification.

Synthesis of Bromide **12**

*N*-Bromosuccinimide (8.4 g, 47.2 mmol) was added in small portions to a solution of PPh_3_ (12.2 g, 46.5 mmol) in dry DCM (46 mL) under ice-water. The mixture was cooled to r.t. and stirred for 30 min. Then, pyridine (2 mL) and nopol (4.0 g, 24.1 mmol) were successively added to the reaction, and the resulting mixture was stirred overnight at room temperature. Then, the mixture was diluted with hexane and filtered through a silica gel plug. The residue was washed thoroughly three times with EtOAc:hexane (6:6 mL) by stirring for around 1 h and filtered through the silica gel plug again. The solution was concentrated in vacuo. The product was isolated by flash column chromatography (hexane) to obtain corresponding bromide as a colorless oil (5.2 g, 94%). Spectroscopic data were consistent with those previously reported [42].

Synthesis of Phthalimides **3**, **9** and **13**

A mixture containing appropriate bromide (10.2 mmol) and potassium phthalimide (10.2 mmol) in 20 mL of dry DMF was stirred at 60 °C until completion of the reaction. Then, the reaction mixture was cooled to r.t. and water was added. The product was extracted with EtOAc. The combined organic phase was successively washed with water and brine and dried over Na_2_SO_4_. The product was purified by column chromatography on silica gel (hexane/EtOAc).

2-(((1*R*)-6,6-Dimethylbicyclo [3.1.1]hept-2-en-2-yl)methyl)isoindoline-1,3-dione **3**

^1^H NMR (300 MHz, CDCl_3_) δ 0.74 (s, 3H), 1.15 (d, *J* = 8.6 Hz, 1H), 1.21 (s, 3H), 2.00–2.07 (m, 1H), 2.08–2.15 (m, 1H), 2.17–2.26 (m, 2H), 2.28–2.39 (m, 1H), 4.08–4.24 (m, 2H), 5.39–5.44 (m, 1H), 7.66–7.74 (m, 2H), 7.79–7.88 (m, 2H). ^13^C NMR (151 MHz, CDCl_3_) δ 168.1, 142.1, 133.8, 132.0, 123.1, 119.7, 43.8, 42.3, 40.6, 38.0, 31.3, 31.0, 26.0, 20.8. ${\left[\alpha \right]}_{D}^{24.9}$ = −28.4 (c 0.56 in CHCl_3_).

Spectroscopic data of compound **13** were consistent with those previously reported [42].

Synthesis of Amines **4**, **10** and **14**

A solution of appropriate phthalimide (3.6 mmol) and ethylenediamine (0.8 mL, 12.0 mmol) in 11 mL of MeOH was refluxed for 8 h. Then, the reaction mixture was cooled to r.t. and the solvent was evaporated. Hexane was added to the residue. The solid was filtered, and the hexane solution was washed with water and brine and dried over Na_2_SO_4_. The crude products were used in the next step without purification.

Synthesis of methyl 4-formylbenzoate **15**

Compound **15** was synthesized as described in [43]. Spectroscopic data were consistent with those previously reported [44].

Synthesis of (*E*)-3-(4-methoxycarbonylphenyl)prop-2-enoic Acid **16**

Compound **16** was synthesized as described in [43]. Spectroscopic data were consistent with those previously reported [45].

Synthesis of (*E*)-4-(3-methoxy-3-oxoprop-1-en-1-yl)benzoic Acid **19**

Compound **19** was synthesized as described in [43]. Spectroscopic data were consistent with those previously reported [46].

Synthesis of Esters **17a**–**c** and **20a**–**c**

To a mixture of (*E*)-3-(4-methoxycarbonylphenyl)prop-2-enoic acid (4.9 mmol), amine (5.3 mmol) and pyridine (1.3 mL) in ethyl acetate (2.7 mL), T3P (*n*-propanephosphonic acid anhydride, 50 wt.% solution in ethyl acetate, 10 mmol) was added. The resulting solution was stirred at 75 °C for 8–10 h and cooled to r.t., and then a saturated solution of sodium bicarbonate was added. The precipitated solid was filtered, washed with water and dried.

Methyl 4-((*E*)-3-((((1*R*)-6,6-Dimethylbicyclo[3.1.1]hept-2-en-2-yl)methyl)amino)-3-oxoprop-1-en-1-yl)benzoate **17a**

White Solid, Yield 61%, m.p. 87.1–88.6 °C

^1^H NMR (400 MHz, CDCl_3_) δ 0.83 (s, 3H), 1.16 (d, *J* = 8.7 Hz, 1H), 1.26 (s, 3H), 2.04–2.12 (m, 2H), 2.16–2.34 (m, 2H), 2.38 (dt, *J* = 8.6, 5.6 Hz, 1H), 3.80–4.00 (m, 5H), 5.39–5.46 (m, 1H), 5.53–5.62 (m, 1H), 6.45 (d, *J* = 15.6 Hz, 1H), 7.54 (d, *J* = 8.4 Hz, 2H), 7.63 (d, *J* = 15.6 Hz, 1H), 8.01 (d, *J* = 8.4 Hz, 2H). ^13^C NMR (126 MHz, CDCl_3_) δ 166.4, 165.2, 144.1, 139.6, 139.1, 130.6, 129.9, 127.5, 122.9, 118.7, 52.1, 44.3, 43.9, 40.5, 37.9, 31.4, 31.0, 25.9, 21.0. HRMS: *m*/*z* 339.1832 (M^+^ C_21_H_25_O_3_N_1_^+^, calc. 339.1829). ${\left[\alpha \right]}_{D}^{25}$ = −17.0 (c 0.74 in CHCl_3_).

Methyl 4-((*E*)-3-((((1*S*)-6,6-Dimethylbicyclo[3.1.1]hept-2-en-2-yl)methyl)amino)-3-oxoprop-1-en-1-yl)benzoate **17b**

White Solid, Yield 79%

NMR spectra were identical to **17a**. HRMS: *m*/*z* 339.1825 (M^+^ C_21_H_25_O_3_N^+^, calc. 333.1829). ${\left[\alpha \right]}_{D}^{25}$ = +24.1 (c 0.43 in CHCl_3_).

Methyl 4-((*E*)-3-((2-((1*R*)-6,6-Dimethylbicyclo[3.1.1]hept-2-en-2-yl)ethyl)amino)-3-oxoprop-1-en-1-yl)benzoate **17c**

White Solid, Yield 74%, m.p. 104.0–105.2 °C

^1^H NMR (500 MHz, CDCl_3_) δ 0.81 (s, 3H), 1.10 (d, *J* = 8.6 Hz, 1H), 1.24 (s, 3H), 2.01–2.12 (m, 2H), 2.15–2.31 (m, 4H), 2.36 (dt, *J* = 8.7, 5.6 Hz, 1H), 3.30–3.54 (m, 2H), 3.89 (s, 3H), 5.25–5.36 (m, 1H), 5.75 (t, *J* = 5.6 Hz, 1H), 6.43 (d, *J* = 15.6 Hz, 1H), 7.52 (d, *J* = 8.0 Hz, 2H), 7.60 (d, *J* = 15.6 Hz, 1H), 7.98 (d, *J* = 8.0 Hz, 2H).^13^C NMR (126 MHz, CDCl_3_) δ 166.4, 165.0, 145.1, 139.4, 139.0, 130.6, 129.9, 127.5, 122.9, 118.7, 52.1, 45.0, 40.5, 37.8, 37.1, 36.2, 31.6, 31.2, 26.0, 21.1. HRMS: *m*/*z* 353.1979 (M^+^ C_22_H_27_O_3_N_1_^+^, calc. 353.1986). ${\left[\alpha \right]}_{D}^{25.5}$ = −13.0 (c 0.4 in CHCl_3_).

Methyl (*E*)-3-(4-((((1*R*)-6,6-Dimethylbicyclo[3.1.1]hept-2-en-2-yl)methyl)carbamoyl)phenyl)acrylate **20a**

White Solid, Yield 76%, m.p. 100.5–101.7 °C

^1^H NMR (400 MHz, CDCl_3_) δ 0.82 (s, 3H), 1.16 (d, *J* = 8.7 Hz, 1H), 1.25 (s, 3H), 2.05–2.12 (m, 2H), 2.15–2.33 (m, 2H), 2.37 (dt, *J* = 8.7, 5.6 Hz, 1H), 3.78 (s, 3H), 3.94 (dq, *J* = 5.6, 1.8 Hz, 2H), 5.41–5.47 (m, 1H), 6.14–6.23 (m, 1H), 6.46 (d, *J* = 16.0 Hz, 1H), 7.53 (d, *J* = 8.4 Hz, 2H), 7.66 (d, *J* = 16.1 Hz, 1H), 7.75 (d, *J* = 8.5 Hz, 2H). ^13^C NMR (101 MHz, CDCl_3_) δ 166.9, 166.4, 144.2, 143.3, 137.1, 135.9, 128.0, 127.3, 119.5, 118.9, 51.7, 44.6, 44.0, 40.6, 37.9, 31.4, 31.0, 26.0, 21.0. HRMS: *m*/*z* 339.1827 (M^+^ C_21_H_25_O_3_N_1_^+^, calc. 339.1829). ${\left[\alpha \right]}_{D}^{25}$ = −22.8 (c 0.67 in CHCl_3_).

Methyl (*E*)-3-(4-((((1*S*)-6,6-Dimethylbicyclo [3.1.1]hept-2-en-2-yl)methyl)carbamoyl)phenyl)acrylate **20b**

White Solid, Yield 73%

NMR spectra were identical to **20a.** HRMS: *m*/*z* 339.1827 (M^+^ C_21_H_25_O_3_N^+^, calc. 333.1829). ${\left[\alpha \right]}_{D}^{25}$ *=* +26.0 (c 0.40 in CHCl_3_).

Methyl (*E*)-3-(4-((2-((1*R*)-6,6-Dimethylbicyclo[3.1.1]hept-2-en-2-yl)ethyl)carbamoyl)phenyl)acrylate **20c**

White Solid, Yield 69%, m.p. 98.2–99.4 °C

^1^H NMR (500 MHz, CDCl_3_) δ 0.82 (s, 3H), 1.10 (d, *J* = 8.6 Hz, 1H), 1.25 (s, 3H), 2.04–2.13 (m, 2H), 2.16–2.32 (m, 4H), 2.37 (dt, *J* = 8.7, 5.6 Hz, 1H), 3.39–3.53 (m, 2H), 3.79 (s, 3H), 5.34 (dt, *J* = 3.0, 1.6 Hz, 1H), 6.17 (t, *J* = 5.3 Hz, 1H), 6.47 (d, *J* = 16.1 Hz, 1H), 7.55 (d, *J* = 8.3 Hz, 2H), 7.67 (d, *J* = 16.0 Hz, 1H), 7.72 (d, *J* = 8.3 Hz, 2H).^13^C NMR (126 MHz, CDCl_3_) δ 167.0, 166.3, 145.2, 143.4, 137.0, 135.9, 128.1, 127.2, 119.4, 118.9, 51.8, 45.0, 40.5, 37.8, 37.2, 36.2, 31.7, 31.2, 26.0, 21.1. HRMS: *m*/*z* 353.1985 (M^+^ C_22_H_27_O_3_N_1_^+^, calc. 353.1986). ${\left[\alpha \right]}_{D}^{25.5}$ = −10.7 (c 0.34 in CHCl_3_).

Synthesis of Hydroxamic Acids **18a**–**c** and **21a**–**c**

KOH (0.6 g, 10.9 mmol) was added to a cooled suspension of NH_2_OH × HCl (0.5 g, 7.3 mmol in 3 mL of MeOH). The resulting mixture was vigorously stirred at room temperature for 30 min and the solid that formed was filtered. The resulting filtrate was added to a solution of an ester (0.3 mmol) in 1 mL of MeOH cooled by an ice-water bath. The mixture was stirred at 0 °C for 10 min, and the solvent was evaporated under reduced pressure. Water was added to the residue, and the solution formed was neutralized with aqueous HCl to pH 5–6. The solid that precipitated was filtered, washed with water and dried. The product was recrystallized from EtOH.

4-((*E*)-3-((((1*R*)-6,6-Dimethylbicyclo[3.1.1]hept-2-en-2-yl)methyl)amino)-3-oxoprop-1-en-1-yl)-*N*-hydroxybenzamide **18a**

White Solid, Yield 59%, m.p. 96.5 °C

^1^H NMR (400 MHz, DMSO-*d*_6_) δ 0.79 (s, 3H), 1.10 (d, *J* = 8.4 Hz, 1H), 1.24 (s, 3H), 1.99–2.08 (m, 2H), 2.09–2.30 (m, 2H), 2.35 (dt, *J* = 8.4, 5.5 Hz, 1H), 3.61–3.83 (m, 2H), 5.35 (s, 1H), 6.75 (d, *J* = 15.7 Hz, 1H), 7.45 (d, *J* = 15.7 Hz, 1H), 7.62 (d, *J* = 8.0 Hz, 2H), 7.78 (d, *J* = 8.0 Hz, 2H), 8.21 (t, *J* = 5.9 Hz, 1H), 9.10 (s, 1H), 11.29 (s, 1H). ^13^C NMR (101 MHz, DMSO-*d*_6_) δ 164.6, 163.7, 145.0, 137.7, 137.6, 133.2, 127.5, 127.5, 123.8, 116.7, 43.4, 43.2, 40.3, 37.6, 31.1, 30.7, 26.0, 20.9. HRMS: *m*/*z* 340.1783 (M^+^ C_20_H_24_O_3_N_2_^+^, calc. 340.1781). ${\left[\alpha \right]}_{D}^{25}$ = −23.1 (c 0.4 in MeOH).

4-((*E*)-3-((((1*S*)-6,6-Dimethylbicyclo[3.1.1]hept-2-en-2-yl)methyl)amino)-3-oxoprop-1-en-1-yl)-*N*-hydroxybenzamide **18b**

White Solid, Yield 64%

NMR spectra were identical to **18a**. HRMS: *m*/*z* 340.1783 (M^+^ C_20_H_24_O_3_N_2_^+^, calc. 340.1781). ${\left[\alpha \right]}_{D}^{25}$ = +21.0 (c 0.42 in CHCl_3_).

4-((*E*)-3-((2-((1*R*)-6,6-Dimethylbicyclo[3.1.1]hept-2-en-2-yl)ethyl)amino)-3-oxoprop-1-en-1-yl)-*N*-hydroxybenzamide **18c**

White Solid, Yield 73%, m.p. 189.7–191.1 °C

^1^H NMR (500 MHz, DMSO-*d*_6_) δ 0.79 (s, 3H), 1.09 (d, *J* = 8.4 Hz, 1H), 1.25 (s, 3H), 1.95–2.26 (m, 6H), 2.30–2.39 (m, 1H), 3.12–3.25 (m, 2H), 5.27 (s, 1H), 6.70 (d, *J* = 15.8 Hz, 1H), 7.42 (d, *J* = 15.8 Hz, 1H), 7.63 (d, *J* = 8.0 Hz, 2H), 7.77 (d, *J* = 8.0 Hz, 2H), 8.11 (t, *J* = 5.7 Hz, 1H), 9.13 (s, 1H), 11.31 (s, 1H).^13^C NMR (126 MHz, DMSO-*d*_6_) δ 164.6, 163.7, 145.4, 137.6, 137.5, 133.2, 127.5, 123.9, 117.2, 45.0, 40.1, 37.6, 37.2, 36.4, 31.2, 30.9, 26.1, 21.1. HRMS: *m*/*z* 354.1932 (M^+^ C_21_H_26_O_3_N_2_^+^, calc. 354.1938). ${\left[\alpha \right]}_{D}^{25.5}$ = −5.6 (c 0.46 in MeOH).

*N*-(((1*R*)-6,6-Dimethylbicyclo[3.1.1]hept-2-en-2-yl)methyl)-4-((*E*)-3-(hydroxyamino)-3-oxoprop-1-en-1-yl)benzamide **21a**

White Solid, Yield 67%, m.p. 74.6 °C

^1^H NMR (500 MHz, DMSO-*d*_6_) δ 0.79 (s, 3H), 1.11 (d, *J* = 8.4 Hz, 1H), 1.23 (s, 3H), 2.00–2.07 (m, 1H), 2.07–2.11 (m, 1H), 2.11–2.30 (m, 2H), 2.30–2.41 (m, 1H), 3.68–3.90 (m, 2H), 5.34 (s, 1H), 6.54 (d, *J* = 15.8 Hz, 1H), 7.49 (d, *J* = 15.8 Hz, 1H), 7.64 (d, *J* = 7.9 Hz, 2H), 7.86 (d, *J* = 7.9 Hz, 2H), 8.63 (t, *J* = 6.0 Hz, 1H), 9.13 (s, 1H), 10.84 (s, 1H). ^13^C NMR (126 MHz, DMSO-*d*_6_) δ 165.8, 162.7, 145.4, 137.7, 137.6, 135.4, 128.1, 127.6, 120.9, 116.8, 43.7, 43.6, 40.6, 37.9, 31.3, 30.9, 26.4, 21.2. HRMS: *m*/*z* 340.1785 (M^+^ C_20_H_24_O_3_N_2_^+^, calc. 340.1781). ${\left[\alpha \right]}_{D}^{25}$ = −21.9 (c 0.4 in MeOH).

*N*-(((1*S*)-6,6-Dimethylbicyclo[3.1.1]hept-2-en-2-yl)methyl)-4-((*E*)-3-(hydroxyamino)-3-oxoprop-1-en-1-yl)benzamide **21b**

White Solid, Yield 52%

NMR spectra were identical to **21a.** HRMS: *m*/*z* 340.1780 (M^+^ C_20_H_24_O_3_N_2_^+^, calc. 340.1781). ${\left[\alpha \right]}_{D}^{25}$ = +22.3 (c 0.48 in CHCl_3_).

*N*-(2-((1*R*)-6,6-Dimethylbicyclo [3.1.1]hept-2-en-2-yl)ethyl)-4-((*E*)-3-(hydroxyamino)-3-oxoprop-1-en-1-yl)benzamide **21c**

White Solid, Yield 70%, m.p. 132.5–132.6 °C

^1^H NMR (300 MHz, DMSO-*d*_6_) δ 0.91 (s, 3H), 1.20 (d, *J* = 8.4 Hz, 1H), 1.36 (s, 3H), 2.17 (d, *J* = 5.6 Hz, 2H), 2.23–2.39 (m, 4H), 2.45 (dt, *J* = 8.4, 5.6 Hz, 1H), 3.38 (q, *J* = 7.3, 6.6 Hz, 2H), 5.40 (s, 1H), 6.64 (d, *J* = 15.8 Hz, 1H), 7.58 (d, *J* = 15.8 Hz, 1H), 7.74 (d, *J* = 8.1 Hz, 2H), 7.93 (d, *J* = 8.1 Hz, 2H), 8.55 (t, *J* = 5.6 Hz, 1H). ^13^C NMR (101 MHz, DMSO-*d*_6_) δ 165.5, 162.5, 145.6, 137.5, 137.4, 135.2, 127.8, 127.4, 120.7, 117.1, 45.2, 40.2, 37.7, 37.6, 36.3, 31.3, 31.0, 26.2, 21.1. HRMS: *m*/*z* 354.1944 (M^+^ C_21_H_26_O_3_N_2_^+^, calc. 354.1938). ${\left[\alpha \right]}_{D}^{25.5}$ = −7.6 (c 0.48 in MeOH).

### 2.2. HDAC Inhibitory Activity Assay

HDAC1-inhibitory activity was determined using the HDAC activity analysis kit by fluorescence measurement (HDAC1 Fluorimetric Drug Discovery Assay Kit A Fluoro de Lys Fluorescent Assay System, Enzo Life Sciences International, Inc., Farmingdale, New York, USA) in accordance with the manufacturer’s instructions. Fluorescence was measured at an excitation wavelength of 360 nm and emission of 460 nm. The well-known histone deacetylase inhibitor Trichostatin A was used as a reference compound. All experiments were carried out in three repetitions.

### 2.3. Cell Culture

In this study, tumor cell cultures of human cervical carcinoma (HeLa) and ovarian teratocarcinoma (PA-1) were used, as well as conditionally normal cells obtained from human embryonic kidney (Hek-293) purchased from the Federal State Budgetary Institution of Science Institute of Cytology of the Russian Academy of Sciences. For all experiments using cell lines, cells were cultured in a DMEM culture medium with the addition of 10% fetal bovine serum, 2 mM glutamine and 1% penicillin-streptomycin as antibiotics under standard conditions at 37 °C with 5% CO_2_.

To obtain a cisplatin-resistant cell line of human cervical carcinoma HeLa/CDDP, when the “native” HeLa cell culture reached 70% confluence, the procedure was started by “step-by-step” increases in the cytostatic doses. To perform this, every 3–4 days, the cells were treated with low concentrations of cytostatic (0.5–1–5–10–15–25 µM). Each dose of cisplatin was administered 7 times, presowing the culture.

### 2.4. Glycolysis Flux Assay

In order to achieve a 90–95% monolayer, approximately 40,000 PA-1 cells per well were placed in XF96 cell-culture plates and a glycolysis stress test was performed 24 h later using an XFe96 extracellular flow analyzer (Seahorse Bioscience, North Billerica, MA, USA) by measuring the rate of extracellular medium acidification (ECAR).

Briefly, after the cells were seeded, the plate was placed in an incubator and cultured for 24 h under the standard conditions described above. After the incubation time, the nutrient medium was aspirated, and the cells were washed and placed in an XF-analysis medium that did not contain serum, glutamine, pyruvate and glucose, and they were incubated for another 60 min under conditions without maintaining the CO_2_ level at 37 °C.

After placing the tablet in the analyzer, a step-by-step measurement of the extracellular acidification rate of the medium was performed in response to the sequential addition of modulators: port A—the studied compounds at a concentration of 100 µM, port B—10 mM of glucose (Seahorse Bioscience, North Billerica, MA, USA), port C—1 µM of oligomycin (Sigma Aldrich, St. Louis, MI, USA) and port D—25 mM 2-deoxy-D-glucose (Sigma Aldrich, St. Louis, MI, USA) [47]. To ensure the stability of each measurement, three separate readings were taken.

Glycolysis was measured by estimating the difference between the maximum ECAR after glucose injection and the last measurement before glucose addition. Such a parameter of glycolytic function as glycolytic capacity was determined by analyzing the difference between the maximum ECAR after oligomycin injection and the last measurement before glucose addition. Glycolytic reserve was defined as the difference between glycolytic capacity and glycolysis.

### 2.5. Assay of Cell Death

Cell viability was measured using MTT analysis, as described earlier [48]. In short, 10,000 HeLa and Hek-293 cells per well were placed in sterile 96-well culture plates and cultured under the standard conditions described above for 24 h to adhere. After the time elapsed, the cells were treated with the studied compounds in a concentration range from 0.1 to 100 µM (0.1–1–3–10–30–100) and incubated for 24 h. The control samples contained an equivalent volume of solvent (DMSO < 1%). At the end of incubation, cell viability was measured by adding an MTT solution (the final concentration was 5 mg/mL, DiaM, Moscow, Russia). After 2 h of incubation at 37 °C, the nutrient medium was carefully aspirated and replaced with 200 µL of dimethyl sulfoxide to dissolve the formazane granules. The optical density of the formazane solution was determined using a multifunctional analyzer Cytation™3 (BioTek Instruments, Inc., Winooski, VT, USA) at λ = 530 nm. All experiments were carried out in three repetitions.

### 2.6. Drug Combination Analysis and Combination Index (CI) Calculation

The IC_50_ values of the cytotoxic effect of the test compound and cisplatin were determined using the analysis described above (Section 2.5). Then, the cells were treated with a combination of increasing doses of cytostatic agents and the test compound with a constant ratio of 1:4, and subsequently, a cell death analysis was performed. The CI values were calculated and plotted using CompuSyn.exe (Biosoft, Ferguson, MO, USA) based on the concentration and effect of each agent and their combinations according to instructions of the software. Synergistic, additive and antagonistic effects were defined by CI < 0.75, CI = 0.75–1.25 and CI > 1.25, respectively, as described by [49].

### 2.7. Molecular Docking and ADME Evaluation

Three-dimensional structural models of the selected target proteins were obtained from the RCSB PDB protein data bank (https://www.rcsb.org/, accessed on 19 July 2023)—crystal structures of hexokinase 1 (PDB ID: 4F9O [50]); 6-phosphofructo-2-kinase (PDB ID: 1K6M [51]) and pyruvate kinase M2 (PDB ID: 1T5A [52]). Using UCSF Chimera software (version 1.17.3, (Resource for Biocomputing, Visualization, and Informatics from the University of California, San Francisco, CA, USA)) [53], the geometry of proteins was prepared by removing various ligands (if appropriate structures were used), metal ions, nonkey waters and other nonkey small molecules, as well as by adding hydrogen atoms, charges and missing side chains.

The minimization of the structure of all ligands was carried out using the UCSF Chimera software in the Merck molecular force field MMFF94 [54].

The molecular docking to the allosteric centers of proteins was performed using the AutoDock Vina package (version 1.1.2, (Molecular Graphics Laboratory, The Scripps Research Institute, La Jolla, CA, USA)) [55]. It should be noted that the allosteric regulation of these enzymes takes a central place and makes a significant contribution to the enzyme activity modulation [56,57,58]. The prediction of the interactions in the protein–ligand complexes was carried out on the basis of the criteria of complementarity of the form of the chemical compound with their binding affinity.

2-Deoxy-glucose-6-phosphate and L-phenylalanine were selected as reference molecules (their structures are shown in Figures S1 and S3 (Supplementary Materials)), for which the allosteric inhibitory effects are confirmed in the literature with respect to hexokinase 1 [57,59] and pyruvate kinase M2 [56], respectively.

Visualization and a detailed study of molecular interactions were carried out using the software Biovia Discovery Studio 2021.

ADME descriptors were obtained using software from the Swiss Institute of Bioinformatics, presented on the SwissADME online server [60].

### 2.8. Statistics

The results of all experiments are presented as mean ± SEM. To compare the means of all groups, results were compared by using one-way ANOVA by Dunnett’s post hoc test, with values from 3 independent experiments with triplicate determination. All statistical analyses were conducted using GraphPad Prism 5 (GraphPad Software, San Diego, CA, USA).

## 3. Results and Discussion

### 3.1. Chemistry

**Scheme 1:**
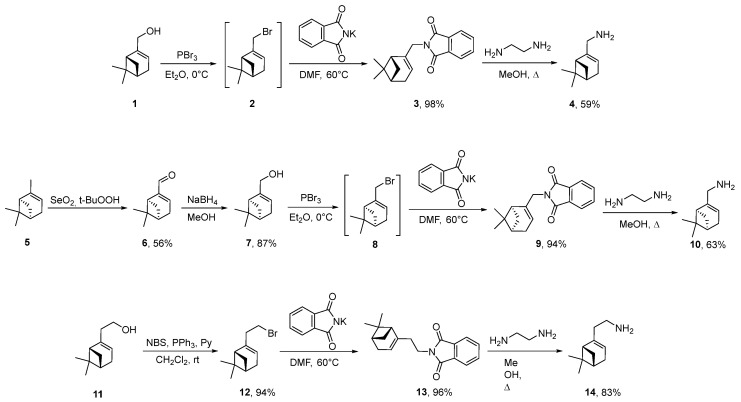
Synthesis of starting monoterpene amines **4**, **10** and **14**.

Initially, a three-step synthesis of (−)-myrtenylamine **4** was carried out, starting from commercially available (−)-myrtenol **1**, which was converted to corresponding bromide **2** using PBr_3_ in Et_2_O (Scheme 1). Then, interaction between compound **2** and potassium phthalimide in DMF at 60 °C gave *N*-alkylsubstituted phthalimide **3** with 98% yield. Refluxing of compound **3** with ethylenediamine in MeOH resulted in (−)-myrtenylamine **4**. To obtain (+)-myrtenylamine **10**, (+)-α-pinene **5** was oxidized using SeO_2_/*t*-BuOOH to (+)-myrtenal **6**, which was treated with NaBH_4_ in MeOH, leading to (+)-myrtenol **7**. Further transformations identical to that of (−)-myrtenol allowed us to synthesize (+)-myrtenylamine **10**. A similar approach was applied to obtain amine **14**. (−)-Nopol **11** was transformed to bromide **12** using NBS and PPh_3_ in CH_2_Cl_2_, which reacted with potassium phthalimide, leading to compound **13**. Ring opening of phthalimide **13** mediated by ethylenediamine gave amine **14**.

**Scheme 2:**
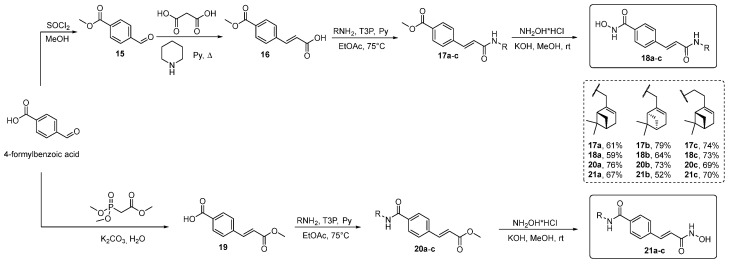
Synthesis of target compounds **18a**–**c** and **21a**–**c**.

For the synthesis of target hydroxamic acids, 4-formylbenzoic acid was treated with SOCl_2_ in MeOH to give corresponding ester **15** (Scheme 2). *para*-Cinnamic acid **16** was obtained by condensation of compound **15** with malonic acid in boiling pyridine in the presence of piperidine. Amides were synthesized by the interaction of acid **16** with monoterpene amines in the presence of *n*-propanephosphonic acid anhydride (T3P) as a coupling reagent to obtain derivatives **17a**–**c**. Hydroxamic acids **18a**–**c** were obtained by the treatment of amides **17a**–**c** with NH_2_OH in MeOH.

In order to synthesize regioisomers **21a**–**c**, the Horner–Wadsworth–Emmons reaction of 4-formylbenzoic acid with trimethyl phosphonoacetate was performed, giving compound **19**. Next, synthetic sequence included amide bond formation, leading to amides **20a**–**c**, followed by NH_2_OH treatment, which resulted in target compounds **21a**–**c**.

### 3.2. Biological Evaluation

Recent advances in basic research into the molecular mechanisms of drug resistance in tumor cells have identified characteristic changes that may be considered to be promising targets for therapeutic intervention [61]. The improperly regulated activity of histone deacetylase, mainly the first isoform, is one of such features leading to the therapeutic resistance development. In particular, in the work of Yang et al. [62], the overexpression of HDACs detected in temozolomide-resistant glioblastoma cells leads to the progression of the cell cycle in the G2/M phase and DNA repair by increasing the levels of the B-cell-specific Mo-MLV 1 (BMI1) integration site and human telomerase reverse transcriptase (hTERT), which support the properties of self-renewal and stemness. In turn, Ding et al. showed that increased HDAC1 levels regulate the sensitivity of the laryngeal squamous cell carcinoma cell line to the action of 5-fluorouracil and cisplatin, increasing the interleukin 8 expression [63]—a critical factor mediating chemoresistance [64]. Being an oncoprotein, HDAC1 also plays a role in the resistance of non-small cell lung cancer cells to gefitinib by reducing the expression of dual-specific phosphatase 1 (DUSP1) [65].

**Figure 2:**
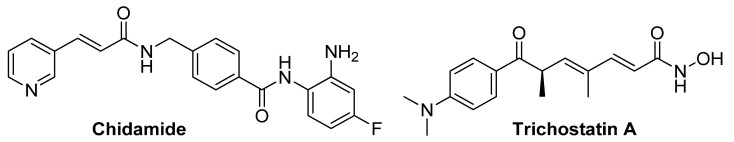
Structural formulas of HDACs inhibitors—modulators of the tumor cell chemoresistance phenomenon.

Thus, targeted inhibition of histone deacetylases is considered to be an effective therapeutic tool for overcoming drug resistance. For example, in the work of Chen and colleagues [66], the use of the HDAC inhibitor panobinostat (Figure 1) significantly increased the apoptotic death of imatinib-resistant cells of chronic myelogenous leukemia-targeting stem cells. The use of the recently FDA-approved HDAC inhibitor Chidamide (Figure 2) on a cell model of primary B-cell lymphoma resistant to rituximab and chemotherapy has shown success by increasing the sensitivity of tumor cells by stimulating cell death through autophagy [67]. And in the work of Sato et al., Trichostatin A (Figure 2) led to the sensibilization of cells of progressive renal cell carcinoma to the sunitinib action due to shifts in cellular metabolism [68].

The hydroxamic acids with a bicyclic pinane backbone synthesized in our work reproduce the above-described model of HDACs inhibitors, which in advance allowed us to assume their prospects as HDACs modulators and led to the study of their HDAC1-inhibitory ability. To confirm the validity of the method, we used Trichostatin A as a reference ligand.

When checking the HDAC1 inhibition profiles of novel hydroxamic acids, it was found that all the studied compounds showed moderate activity against this enzyme (Table 1), as evidenced by the IC_50_ values, which range from 4.45 ± 0.30 (for **18c**) to 8.20 ± 0.49 µM (for **21b**).

And although the synthesized hydroxamic acids failed to achieve the HDAC1-inhibitory effect of Trichostatin A, the discovered phenomenon is positive in the context of our work. Clinically approved HDAC inhibitors, in addition to their positive properties, have significant systemic toxicity in the form of side effects on the healthy microenvironment [69,70]. This is due to the fact that histone deacetylases, in addition to their negative role in various human diseases, also play the role of critical epigenetic regulators during the normal functioning of the body [71]. These enzymes mediate the maintenance of chromatin structure and transcription and, as a consequence, the occurrence of various cellular processes [72]. In turn, moderate inhibition of histone deacetylases suggests the absence of negative effects and their further contribution to the possible manifestation of adjuvant properties. The opening of chromatin configuration induced by HDAC inhibitors due to histone hyperacetylation is expected to increase the accessibility of chromatin to therapeutic agents targeting genomic DNA.

It is also well known that the metabolism of tumor cells is radically different from that of normal cells, which allows them to maintain a high rate of proliferation and prevent apoptotic death [73]. A distinctive feature of cellular metabolism in malignant neoplasms is their extreme dependence on glycolysis [74]. Even in conditions of sufficient oxygen, transformed cells prefer a seemingly less energetically advantageous way of obtaining energy—aerobic glycolysis (2 ATP molecules versus 36 molecules during oxidative phosphorylation). However, to date, it has already been shown that the rate of ATP production through intensive glycolysis in cells of a rapidly proliferating malignant neoplasm can be 100 times higher than with oxidative phosphorylation [75], which explains the expediency of such switching of tumor metabolism. Due to this, transformed cells manage to satisfy their high anabolic needs for intensive proliferation and development [76] and, equally important, to use the metabolic intermediates formed during glycolysis for the biosynthesis of macromolecules that play a critical role in the formation of multidrug resistance [77].

Thus, the experimental observations of recent years increasingly indicate that aberrant glycolysis plays a critical role in the resistance of transformed cells to the action of chemotherapeutic agents [78]. In particular, the analysis of the metabolic profile of tamoxifen-resistant breast cancer cells TamR MCF-7 demonstrated an increased glycolysis rate in contrast to the original cell culture, which correlated with HK2 overexpression [79]—the gene encoding hexokinase 2. Such an increase in the activity of the enzyme that limits the speed of this process led to increased autophagy mediated by the inhibition of mammalian rapamycin target hexokinase 2 (mTOR), due to which tumor cells acquired the property of resistance to tamoxifen. In turn, in the samples obtained from patients with glioblastoma, increased glycolysis was accompanied by the movement of another key enzyme—the M2 pyruvate kinase isoform (PKM2)—into the mitochondria, where phosphorylation and, as a consequence, increased expression of the antiapoptotic protein Bcl-2 [80] occur, which can also be considered to be one of the possible mechanisms of drug-resistance development [81]. Moreover, due to the fact that during intensive glycolysis occurs in neoplastic cells, a large lactate amount is released into the extracellular space and the tumor microenvironment is characterized by acidic pH values [82]. This plays a critical role in the functioning of efflux pumps localized in the cytoplasmic membrane for the outflow of medicinal agents [83], thereby reducing the effectiveness of antitumor agents [84].

Thus, taking into account the important role of glycolysis in the formation of a resistant state in tumor cells to the action of therapeutic agents, targeting this process is considered to be one of the most promising strategies for the creation of medicinal agents for the treatment of malignant neoplasms. In particular, in the work of Wu et al. about the natural flavonol kaempferol, the ability to reverse the resistance of the HCT8-R colorectal carcinoma cell line resistant to the action of 5-fluorouracil was found by targeting PKM2 [85]. A similar effect was shown for the hypoglycemic drug simvastatin, which restores the sensitivity of hepatocellular carcinoma cells LM3-SR to sorafenib, due to the suppression of PKM2-mediated glycolysis [86]. All this confirms the existence of a direct relationship between metabolic reprogramming and the development of resistance to the action of antitumor drugs in neoplastic cells, and allows us to consider the process of glycolysis to be a valuable target for overcoming drug resistance.

Moreover, due to the fact that for some representatives of the hydroxamic acid class, the ability to modulate the glycolytic profile of tumor origin cells was already shown [87], in particular, in the study we published earlier [88], for new hydroxamic acids with a bicyclic pinane backbone, it became advisable to study the inhibitory ability in relation to this process.

To assess the effect of compounds on the bioenergetic profile of human teratocarcinoma PA-1 cell culture, we analyzed the rate of acidification of the medium by cells in real time using a glycolysis stress test [89]. To date, it has been convincingly proven that in epithelial ovarian cancer, deregulation of cellular energetics occurs, mediating the growth, invasion and migration of tumor cells [90,91,92,93].

**Figure 3:**
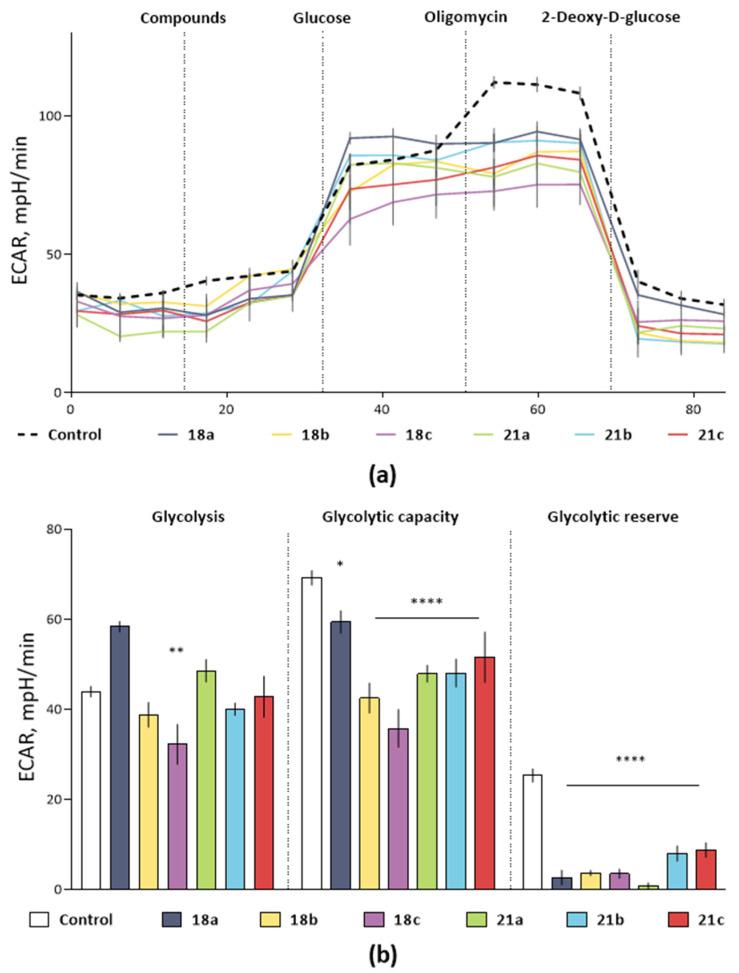
The effect of hydroxamic acids with a bicyclic pinane backbone on the glycolytic profile of PA-1 tumor cells by measuring the rate of medium extracellular acidification (ECAR). (**a**) Kinetic curves of changes in the glycolytic profile under the action of the studied compounds. (**b**) The effect of hydroxamic acids on the main calculated parameters of the PA-1 glycolytic function (glycolysis, glycolytic capacity and glycolytic reserve). The data are presented as a percentage of ECAR relative to the control (DMSO ≤ 1%) as an average value ± SEM. *, ** and ****, *p* < 0.05; *p* < 0.01 and *p* < 0.0001, compared to the control. Statistical analysis was performed using one-way ANOVA followed by Dunnett’s multiple comparison tests. The concentration of the studied compounds was 100 µM, glucose—10 mM, oligomycin—1 µM, 2-fluoro-2-deoxy-D-glucose (2-DG)—25 mM.

As shown in Figure 3, almost all glycolysis parameters were significantly reduced in cells treated with 100 µM of hydroxamic acids, while the most promising inhibition profile was demonstrated for the compound **18c**. Thus, glycolytic capacity equal to the maximum ECAR after oligomycin injection was significantly lower in probes with hydroxamic acid (*p* < 0.0001 vs. control). At the same time, glycolytic reserve, which is the cells’ ability to increase glycolysis to compensate for stressors inside the cell, was inhibited by 86% (*p* = 0.0001 vs. control). Moreover, an essential fact is its pronounced ability to reduce such a critically important parameter of glycolytic function as glycolysis itself by 26.6%, *p* = 0.0061, which was not found for other compounds. Together, these data indicate that **18c** treatment has an extensive impact on the glycolytic pathway.

It should be noted that in order to exclude the possibility of a decrease in ECAR due to cell death under the influence of compounds, and not a specific effect on the glycolytic function of cells, at the end of the glycolysis stress test we performed a cell viability analysis using the MTT test. We did not find any differences in the cell survival of control samples and samples treated with hydroxamic acid (results are not presented). Thus, the results obtained indicate that hydroxamic acids with a bicyclic pinane backbone are negative regulators of aerobic glycolysis, a process that plays a crucial role in the vital activity of tumor cells [76].

To assume the molecular basis of the glycolysis-inhibiting action of hydroxamic acids, we modeled the interaction between the studied compounds and key enzymes of this process by evaluating possible poses of binding compounds with allosteric centers of hexokinase 1 (HK1), 6-phosphofructo-2-kinase (PFK) and pyruvate kinase M2 (PKM2). Thus, the study focused on modeling the binding of six ligands to three target proteins.

As shown in Figures S1–S3 (Supplementary Materials), similar values of the docking score were demonstrated for all the studied compounds and reference ligands in relation to the selected protein targets. And, despite the fact that the ways of interaction of the studied hydroxamic acids differ from those of the reference molecules, the values of the docking score in most cases are equal. It is likely that the structural complexity of these compounds gives rise to additional flexible interactions with target proteins.

**Figure 4:**
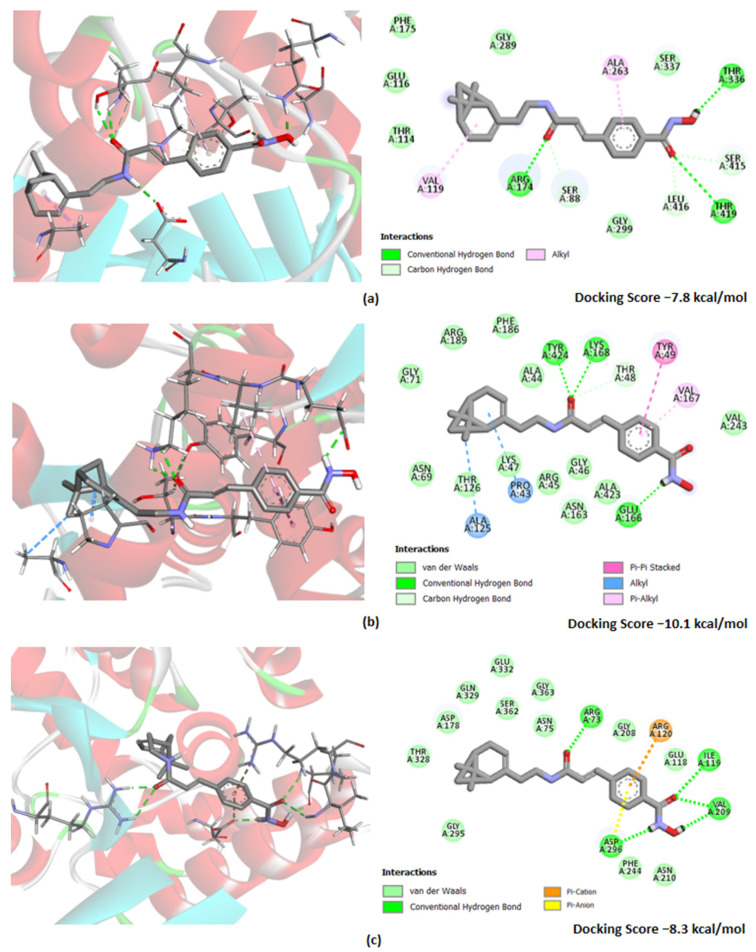
Docking poses of hydroxamic acid **18c** with target proteins: (**a**) hexokinase 1 (PDB ID: 4F9O); (**b**) 6-phosphofructo-2-kinase (PDB ID: 1K6M) and (**c**) pyruvate kinase M2 (PDB ID: 1T5A). Nitrogen and oxygen atoms are colored in blue and red, respectively. The figures on the right side show detailed results reflecting the amino acid residues involved in essential interactions and other significant contacts identified by the “show 2D diagram”. Hydrogen bond (bright green-dotted lines), Van der Waals bond (light green-dotted lines), other interactions (alkyl are shown in light blue-dotted lines; π-alkyl—in light pink-dotted lines; π-π stacked—in bright pink-dotted lines, π-anion and π-cation—yellow and orange-dotted lines, respectively).

As an example, the illustration of the most favorable poses of the **18c** are shown in Figure 4. Two-dimensional representations of the binding mode of other compounds are demonstrated in Figure S1 (Supplementary Materials). As shown in Figure 4a, **18c** exhibited H-bonding with hexokinase at Thr336 (2.37 Å), Thr419 (1.86 Å), Arg174 (1.95 Å), Leu416 (2.38 Å), Ser88 (2.50 Å) and Ser415 (3.03 Å), as well as hydrophobic alkyl bonding at Val119 (4.12 Å) and Ala263 (4.43 Å). On the other hand, it was found that **18c** displayed H-bonding interaction with 6-phosphofructo-2-kinase at Tyr424 (2.11 Å), Lys168 (2.42 Å), Thr48 (2.43 Å) and Glu166 (2.20 Å), as well as other hydrophobic interactions—π-π stacked at Tyr49 (4.66 Å), π-alkyl at Val167 (4.95 Å) and alkyl at Ala125 (4.44 Å) and Pro43 (5.48 Å) (Figure 4b). The docking study of pyruvate kinase M2 with **18c** (Figure 4c) showed hydrogen bonds at Arg73 (2.03 Å), Ile119 (2.51 Å), Val209 (2.49 Å and 1.82 Å) and Asp296 (2.03 Å) at the allosteric center of the enzyme and electrostatic π-cation and π-alkyl bonding—at Arg120 (4.29 Å) and Asp296 (3.50 Å), respectively.

It should also be noted that the docking energy values for **18c** were found to be in the range of −7.8 to −10.1 kcal/mol for all the enzyme targets. Obviously, this may indicate the ability of this hydroxamic acid to inhibit the glycolytic function of cells of tumor origin due to the modulating effect on all key enzymes of this process.

Also, we would like to focus on the most interesting phenomenon. When assessing the affinity of hydroxamic acids to the hexokinase, it was found that **18c** and **21c** have the lowest docking score values, which could indicate a more pronounced affinity of the binding of compounds to the enzyme allosteric center. This may be due to the ability of these ligands to form more hydrogen interactions with HK1, which in this case may have the greatest significance, contributing to a higher affinity to HK1. This is consistent with the lowest value of the docking score function for **18c**, equal to −7.8, which is lower than that of **21c** (−7.5) and other hydroxamic acids (≤−7.2). This may be the key reason for the experimentally detected most effective inhibitory effect on the glycolysis process for **18c** (Figure 3).

Thus, we found that hydroxamic acids cause a metabolic crisis in tumor cells due to the control of glycolysis, possibly mediated by the rate-limiting enzymes HK1, PFK2 and PKM2. Moreover, the better affinity to HK1 suggested by computational methods for the leader, **18c**, may explain the most pronounced glycolysis-inhibiting ability when monitoring the metabolism of tumor cells.

At the next stage, to confirm our hypothesis about the possible chemosensitizing properties of hydroxamic acids, we conducted a set of experiments using objects of the cellular level of the organization.

Initially, in order to assess their own toxic effect and select the safe concentration of the studied compounds, we analyzed the viability of cells pretreated with hydroxamic acids for 24 h in a concentration range from 0.1 to 100 µM. This stage is of absolute importance in the search for potential chemosensitizing agents, since one of the most significant characteristics inherent in such agents is the absence of pronounced cytotoxic properties that can serve as an obstacle to overcoming multidrug resistance and contribute to the manifestation of systemic toxicity. In particular, attempts to introduce the well-known histone deacetylase inhibitors Trichostatin A and vorinostat into clinical practice as adjuvant therapy have so far failed due to their side effects.

Human cervical carcinoma HeLa cells, which are the most optimized cell line for many areas of biological research, were used as a model system [94]. In turn, conditionally normal origin Hek-293 cells, obtained from a human embryonic kidney, served as an object necessary for extrapolating the results to a healthy microenvironment.

As shown in Table 2, the range of IC_50_ values of the cytotoxic effect of hydroxamic acids varies from 18.01 ± 0.47 µM (in the case of **21a**) to 43.59 ± 1.02 µM (for **18b**). Such IC_50_ values are not representative for anticancer drugs, since this indicator is in the nano- or sub-micromolar range for the majority of oncolytics that exist today. However, as mentioned above, the effect we found can be considered to be a positive property for adjuvant agents.

It is also worth noting that we found no differences in the IC_50_ values of the cytotoxic effect between cells of tumor and normal origin. Thus, the range of IC_50_ values on the Hek-293 cell line ranged from 21.19 ± 1.66 µM (in the case of **21a**) to 46.73 ± 2.93 µM (for **18a**). Obviously, such a level of cytotoxicity of hydroxamic acids is unlikely to lead to side effects on a healthy microenvironment. Thus, the absence of increased toxic effects on cells of normal origin determines the expediency of continuing experiments aimed at studying the adjuvant properties of compounds.

Due to the treatment of HeLa with the studied compounds in concentrations up to 5 µM having little or no effect on cellular viability, this concentration of hydroxamic acids was chosen to determine whether the compounds under study could increase the HeLa cell line sensitivity to death induced by the well-known antineoplastic alkylating agent cisplatin. The expediency of choosing this cytostatic drug is due to two fundamental factors. First of all, this is due to the fact that, despite the excellent cisplatin antitumor properties, it is well known in clinical practice that therapeutic failures are associated with the emergence of drug resistance to this cytostatic agent [95,96,97,98], which makes the search for new chemosensitizing agents a promising direction. Moreover, being inhibitors of histone deacetylases, hydroxamic acids are more likely to exert their adjuvant effect precisely against drugs whose mechanism of action is to disrupt the DNA function. This is due to the fact that HDACi promote chromatin relaxation and DNA structure opening, providing unhindered access to chemotherapeutic agents [99].

Thus, the combination of hydroxamic acids with cisplatin was analyzed on the HeLa cervical carcinoma cell line. As shown in Table 3, the IC_50′_s cytotoxic effect during 24 h cisplatin incubation was 28.97 ± 2.80 µM. In turn, the study of the effect of binary combinations of chemical agents on cell survival showed a synergistic effect with bolus administration of 5 µM of **18c** with cytostatic. This was expressed in a significant decrease in the value of cisplatin IC_50_ by 34.50% (*p* = 0.0300). The tendency to display similar properties, but to a lesser extent, was shown by the regioisomer **18c**, hydroxamic acid with an inverted arrangement of the Cap group and the hydroxamate function **21c**.

Thus, supporting the concept of a possible contribution of HDAC1-inhibitory ability to the adjuvant properties, we observed that the hydroxamic acid **18c**, containing a pinane fragment in the Cap group, potentiated the antitumor effect of cytostatics, leading to a better therapeutic result on HeLa cells.

**Figure 5:**
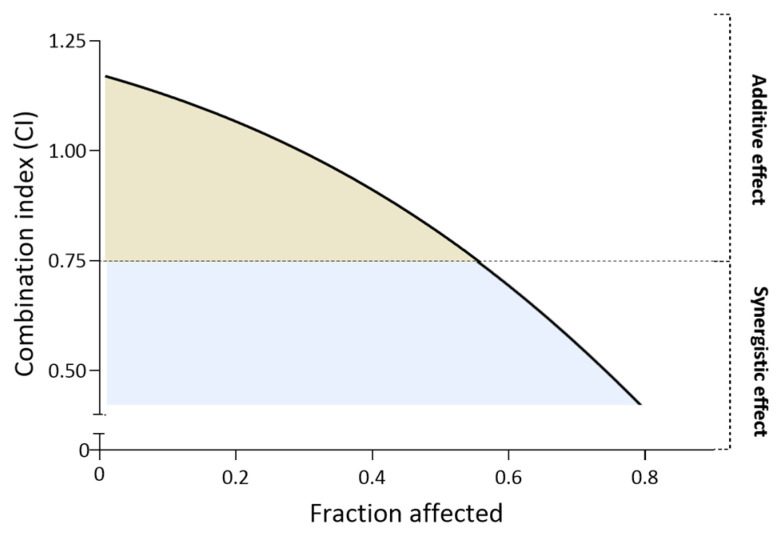
Fraction-CI analysis by CompuSyn.exe showing the nature of the potentiating effect of **18c**.

Then, we calculated the combination index (CI) score to evaluate the combined effect of hydroxamic acid and cisplatin. The fraction-CI analysis indicated that CI values were in two ranges depending on the concentrations used (Figure 5). Thus, at relatively low concentrations, combinations of cisplatin (0.1–10 µM) and **18c** (0.025–2.5 µM) exhibited an additive effect (CI from 0.94 to 1.23) and demonstrated fairly low cytotoxic activity. In turn, when cells were treated with moderate concentrations of cisplatin (15–100 µM) and **18c** (3.75–25 µM), a synergistic effect was detected (CI from 0.75 to 0.64). Obviously, the results obtained indicate that the effect of hydroxamic acid discovered above on the cytotoxic properties of cisplatin realizes its effect due to a synergistic effect.

**Figure 6:**
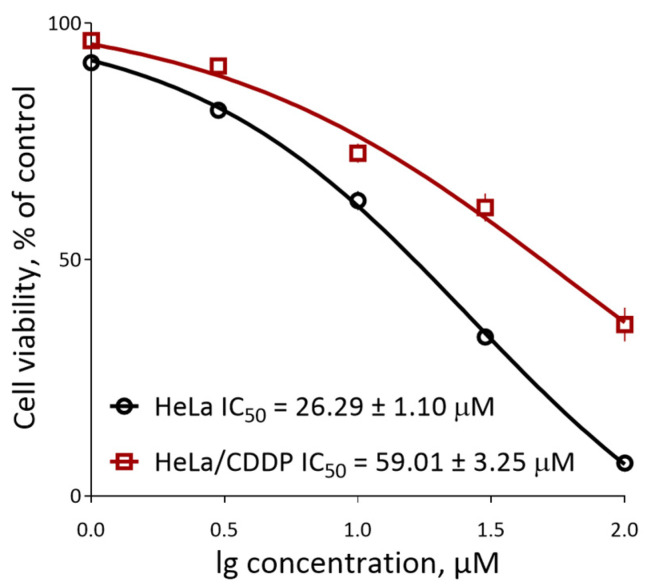
The survival curves of parental HeLa cells and the resistant HeLa/CDDP cells under the action of cisplatin.

To further prove the synergistic effect of hydroxamic acids, we created a cisplatin-resistant cervical carcinoma HeLa cell line, which had significantly reduced sensitivity to various doses of cytostatic (HeLa/CDDP). Thus, treatment of HeLa/CDDP with a concentration equal to the cisplatin detected above IC_50_ barely induced cell death, while the immediate value of semimaximal inhibition in relation to the stable line was increased by more than 50% when compared with parent cells (Figure 6 and Table 4).

These results demonstrate that HeLa/CDDP provides reliable resistance to cisplatin and can serve as a model of chemoresistant tumor cells.

To investigate whether the synergistic cytotoxic effect of the combination of hydroxamic acids and cisplatin occurred in cytostatic-resistant cells, we treated HeLa/CDDP cells with a combination of these compounds at different concentrations of cisplatin along with a fixed dose of 5 μM hydroxamic acids for 24 h.

As shown in Table 4, **18c** is still effective even in cisplatin-resistant HeLa cells. Thus, the addition of low-dose hydroxamic acid resulted in an approximately 2.02-fold decrease in the IC_50_ of cisplatin (29.24 ± 2.08 µM, *p* = 0.9420 when compared with cisplatin for HeLa). Although this hydroxamic acid failed to enhance the initial cytostatic antitumor effect shown on HeLa parent cells, **18c** was able to achieve the key goal of reversing HeLa/CDDP drug resistance.

Thus, hydroxamic acid **18c** with a bicyclic pinane backbone suppressed the viability of human cervical carcinoma cells resistant to cisplatin, which may be due to its synergistic effect with the cytostatic agents.

In addition to the results obtained, we evaluated the pharmacokinetic parameters of the most promising hydroxamic acid, **18c**, with a bicyclic pinane backbone containing a perill fragment in the Cap group using the software SwissAdme free web tool (http://www.swissadme.ch/index.php, accessed on 4 August 2023).

**Figure 7:**
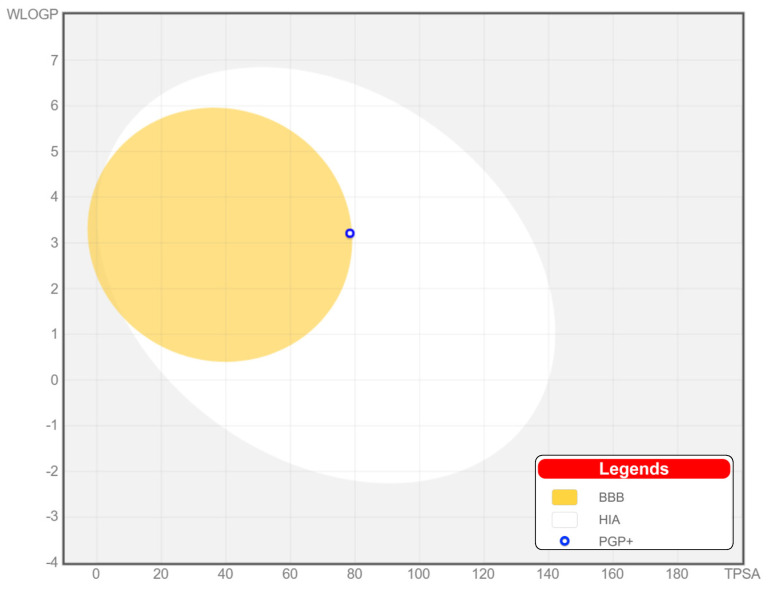
BOILED-Egg model for predicting passive absorption of compounds in the gastrointestinal tract (white area) and penetration into the brain (yellow area).

To visually display the results of the study of the hydroxamic acid **18c**’s pharmacokinetic properties, the prognostic Boiled-Egg model is presented in Figure 7. This model allows one to evaluate the passive gastrointestinal absorption and penetration into the brain of potential drugs by calculating their lipophilicity and polarity.

Based on the calculated data, the value of such a parameter as the topological polar surface area (TPSA), a descriptor used to describe the ability of drugs to penetrate cells through biological barriers, was 78.43 Å^2^. This indicates that **18c** can be positioned for oral use due to the high absorption rate in the human gastrointestinal tract. In addition, the ability to penetrate the blood–brain barrier was also predicted for this compound, as evidenced by **18c**’s presence in the yellow region, which obviously suggests the possibility of its use for therapeutic intervention and for malignant neoplasms of the central nervous system.

Moreover, the results of calculating the values of the main variables of pharmacokinetic characteristics for **18c**, presented in the Table 5, show that this hydroxamic acid fully follows the Lipinski rule, falling within the range of parameters that the drug should have, and also has a good predicted bioavailability index (0.55).

**Figure 8:**
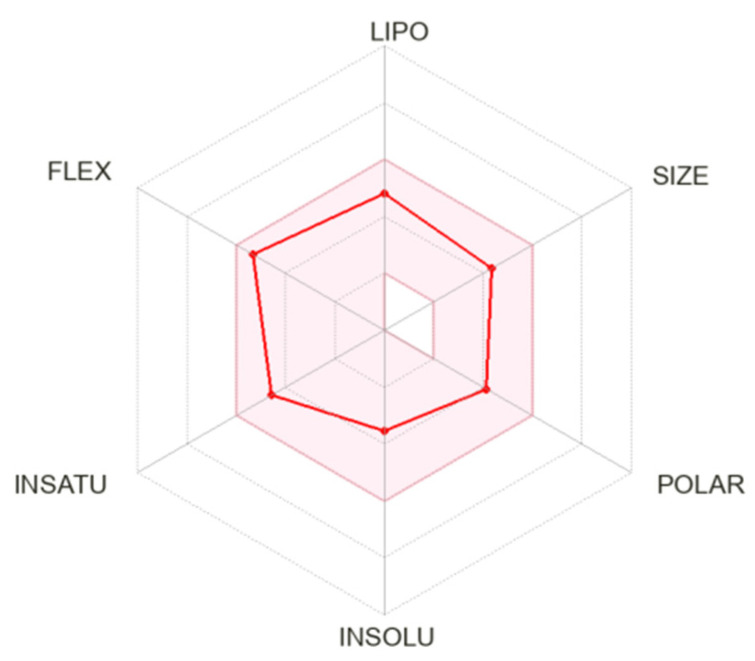
Radar diagram of bioavailability of hydroxamic acid with a bicyclic pinane backbone containing a perill fragment in the Cap group, **18c**. The graphs show the parameters LIPO (lipophilicity), SIZE (molecular weight), POLAR (polarity), INSOLO (insolubility), INSATU (unsaturation) and FLEX (conformational flexibility). The pink-colored zone shows the ideal values of oral bioavailability; the red line shows the direct result for the studied compound.

The obtained results are also clearly presented on the radar bioavailability graph of the studied compound, which displays the assessment of compliance of **18c** with the drug-likeness criteria (Figure 8) for six key physico-chemical properties, including lipophilicity (XLOGP3 from −0.7 to +5.0), size (molecular weight from 150 to 500 g/mol), polarity (TPSA from 20 to 130 Å^2^), solubility (log S is not higher than 6), saturation (the proportion of carbons in sp^3^ hybridization is not less than 0.25) and conformational flexibility (no more than nine rotating bonds) [60].

It was found that **18c** is, in all parameters, included in the radar-colored zone, reflecting the proper physico-chemical characteristics that a bioavailable drug should possess. In other words, based on the data presented in Table 5 and Figure 7 and Figure 8, it can be assumed that hydroxamic acid **18c** has good ADME properties and high bioavailability.

## 4. Conclusions

To date, a critical obstacle to the successful use of chemotherapeutic agents in the treatment of cancer is the acquisition of resistance by transformed cells, which is accompanied by the inactivation of drugs, the occurrence of relapses and the formation of secondary foci of tumor growth. And in view of the growing concern in the scientific community regarding the problem of drug-resistance formation, a large number of research groups are focused on creating unique technological tools that could help overcome chemoresistance and enhance therapeutic responses in the treatment of malignant neoplasms.

In this study, we synthesized a number of hydroxamic acids containing para-substituted cinnamic acid core and bearing bicyclic pinane fragments, including derivatives of (−)-myrtenol, (+)-myrtenol and (−)-nopol, as a Cap-group. The analysis of the biological activity of hydroxamic acid with a bicyclic pinane backbone demonstrated that **18c** synergizes with cisplatin to increase its anticancer effect and overcomes cisplatin resistance. Possible mechanisms of this action could include the histone deacetylase 1 inhibition and glycolytic function. Moreover, a promising pharmacokinetic profile for this hydroxamic acid is predicted.

Thus, a combination of the hydroxamic acids containing a bicyclic pinane backbone and cisplatin may offer a potential therapeutic strategy for the eradication of tumor cells and for overcoming drug resistance in the treatment of cervical carcinoma.

## Figures and Tables

**Table 1:** HDAC1-inhibitory ability of hydroxamic acids with a bicyclic pinane backbone.

Compounds	HDAC1-Inhibitory Ability (IC_50_, µM)	Compounds	HDAC1-Inhibitory Ability (IC_50_, µM)
Trichostatin A	0.22 ± 0.01	**18c**	4.45 ± 0.30
		**21a**	7.23 ± 0.46
**18a**	6.90 ± 0.03	**21b**	8.20 ± 0.49
**18b**	7.96 ± 0.28	**21c**	5.00 ± 0.12

**Table 2:** Values of the IC_50_’s (µM) cytotoxic effect of hydroxamic acids with bicyclic pinane backbone.

Compounds	HeLa	Hek-293	Compounds	HeLa	Hek-293
Trichostatin A	0.38 ± 0.02	0.22 ± 0.01	**18c**	33.81 ± 1.19	34.04 ± 3.04
			**21a**	18.01 ± 0.47	21.19 ± 1.66
**18a**	29.25 ± 1.06	46.73 ± 2.93	**21b**	42.78 ± 1.28	37.19 ± 1.89
**18b**	43.59 ± 1.02	38.85 ± 1.24	**21c**	19.10 ± 0.96	33.86 ± 2.59

**Table 3:** Values of the IC_50′_s (µM) cytotoxic effect of cisplatin in monotherapy and in combination with hydroxamic acids on the human cervical carcinoma HeLa cell line.

Compounds	IC_50_, µM	Compounds	IC_50_, µM
Cisplatin	28.97 ± 2.80	Cisplatin + **18c**	18.98 ± 1.16 *
		Cisplatin + **21a**	27.04 ± 1.44
Cisplatin + **18a**	29.10 ± 1.52	Cisplatin + **21b**	32.69 ± 2.37
Cisplatin + **18b**	30.00 ± 1.56	Cisplatin + **21c**	25.73 ± 2.24

* *p* < 0.05, compared to the cisplatin. Statistical analysis was performed using one-way ANOVA followed by Dunnett’s multiple comparison tests.

**Table 4:** IC_50_ values of the cytotoxic effect of cisplatin in monotherapy and in combination with hydroxamic acids on the resistant cell line of human cervical carcinoma HeLa/CDDP.

Compounds	IC_50_, µM	Compounds	IC_50_, µM
Cisplatin	59.01 ± 3.25	Cisplatin + **18c**	29.24 ± 2.08 **
		Cisplatin + **21a**	59.56 ± 2.74
Cisplatin + **18a**	60.24 ± 3.06	Cisplatin + **21b**	61.55 ± 3.78
Cisplatin + **18b**	60.69 ± 2.26	Cisplatin + **21c**	56.07 ± 1.90

** *p* < 0.01, compared to the cisplatin. Statistical analysis was performed using one-way ANOVA followed by Dunnett’s multiple comparison tests.

**Table 5:** Parameters of ADME and bioavailability of hydroxamic acid with a bicyclic pinane backbone containing a perill fragment in the Cap group, **18c**.

Compound	MW (g/M)	Donors	Acceptors	TPSA	Log *P_o_*_/v_	BA
18c	354.44	3	3	78.43 Å^2^	2.72	0.55

Abbreviations: MW—molecular weight (less than 500 daltons); Donors—number of hydrogen bond donors (no more than 5); Acceptors—number of hydrogen bond acceptors (no more than 10); TPSA—total polar surface area (no more than 150); Log *P*_*o*/v_—octanol–water partition coefficient (not more than more than 5); BA—bioavailability assessment.

## Data Availability

Samples of the compounds and data used during the current study are available from the corresponding author.

## Supplementary Materials

The following supporting information can be downloaded at: https://www.mdpi.com/article/10.3390/cancers15204985/s1. Spectroscopic data of compounds **17a**, **17c**, **18a**, **18c**, **20a**, **20c**, **21a** and **21c**. Figures S1–S3: Molecular docking of ligands against Hexokinase 1, 6-Phosphofructo-2-kinase, Pyruvate kinase M2, respectively.
