# Supplementary material for: Hydroxamic Acids Containing a Bicyclic Pinane Backbone as Epigenetic and Metabolic Regulators: Synergizing Agents to Overcome Cisplatin Resistance
Source: Cancers (Basel). 2023 Oct 14;15(20):4985. doi: 10.3390/cancers15204985 (PMC10605847; doi:10.3390/cancers15204985)

Hydroxamic Acids Containing a Bicyclic Pinane Backbone as Epigenetic and Metabolic Regulators: Synergizing Agents to Overcome Cisplatin Resistance

Aleksandrova Yulia [1], Aldar Munkuev [2], Evgenii Mozhaitsev [2], Evgeniy Suslov [2], Konstantin Volcho [2], Nariman Salakhutdinov [2] and Margarita Neganova [1,*]

[1] Institute of Physiologically Active Compounds at Federal Research Center of Problems of Chemical Physics and Medicinal Chemistry, Russian Academy of Sciences, Severnij Pr. 1, Chernogolovka 142432, Russia

[2] Department of Medicinal Chemistry, N. N. Vorozhtsov Novosibirsk Institute of Organic Chemistry, Siberian Branch, Russian Academy of Sciences, Lavrentiev Ave., 9, Novosibirsk 630090, Russia

* Correspondence: neganovam@ipac.ac.ru; Tel.: +7-(962)-937-68-69

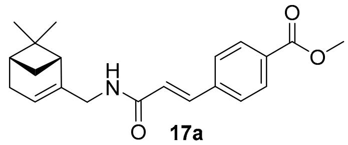

17a

^{1}H NMR spectrum of compound **17a**

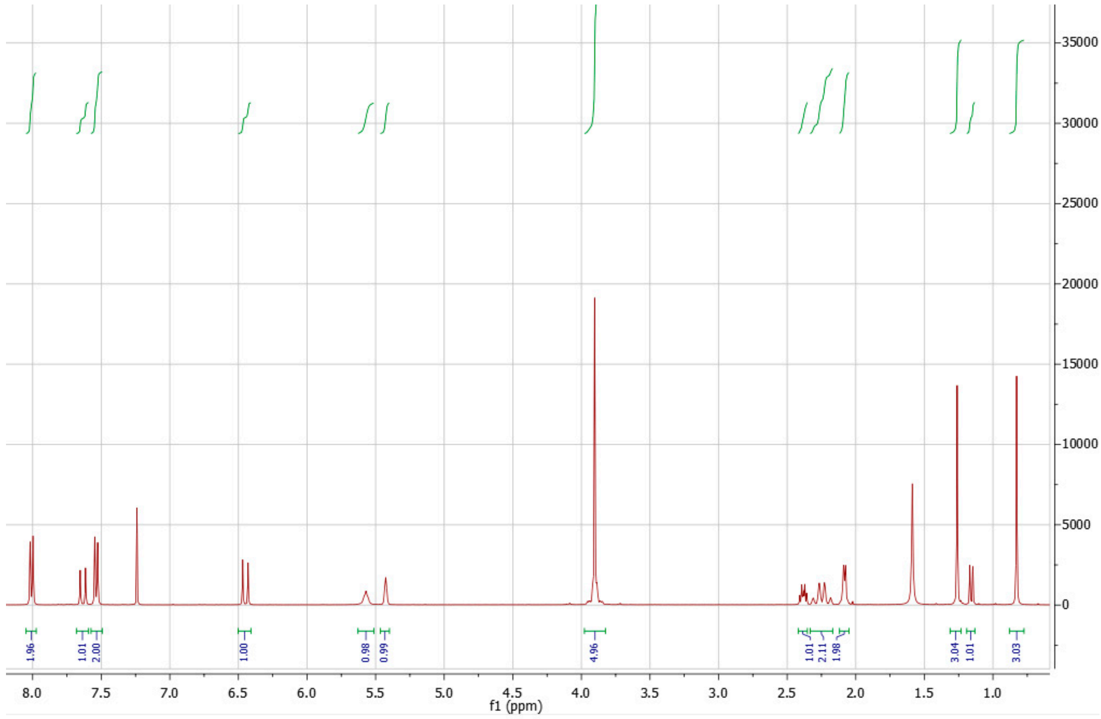

^{13}C NMR spectrum of compound **17a**

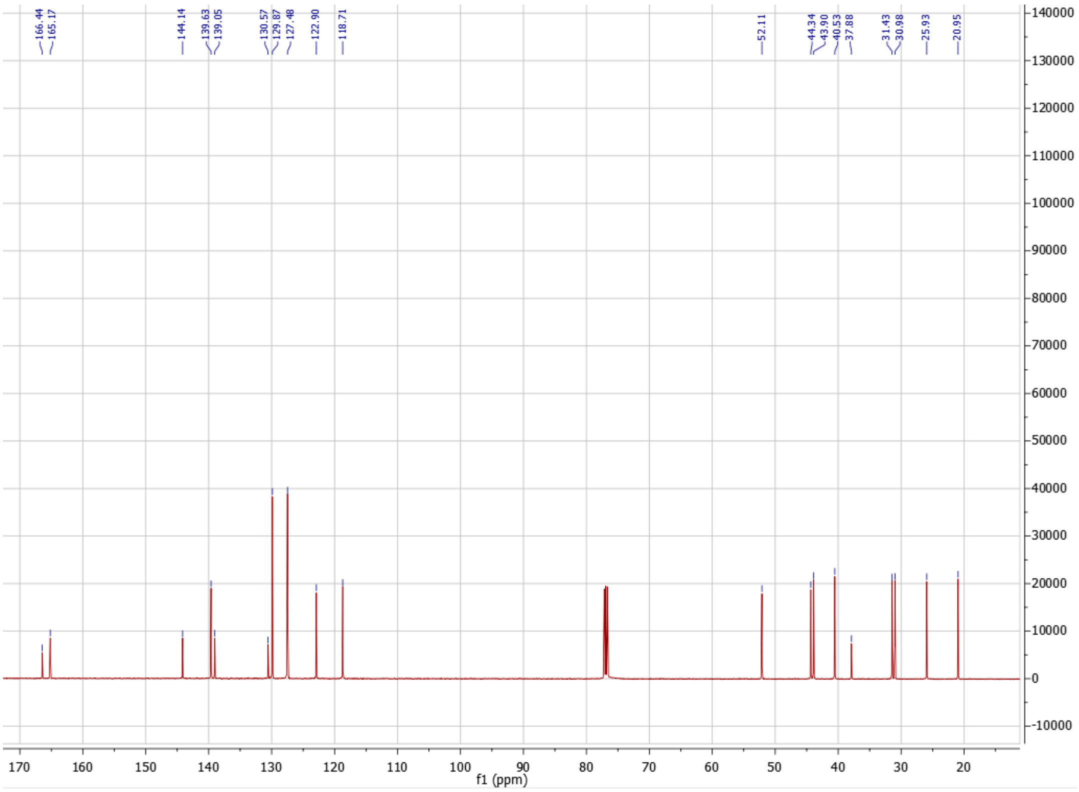

Mass-spectrum of compound **17a**

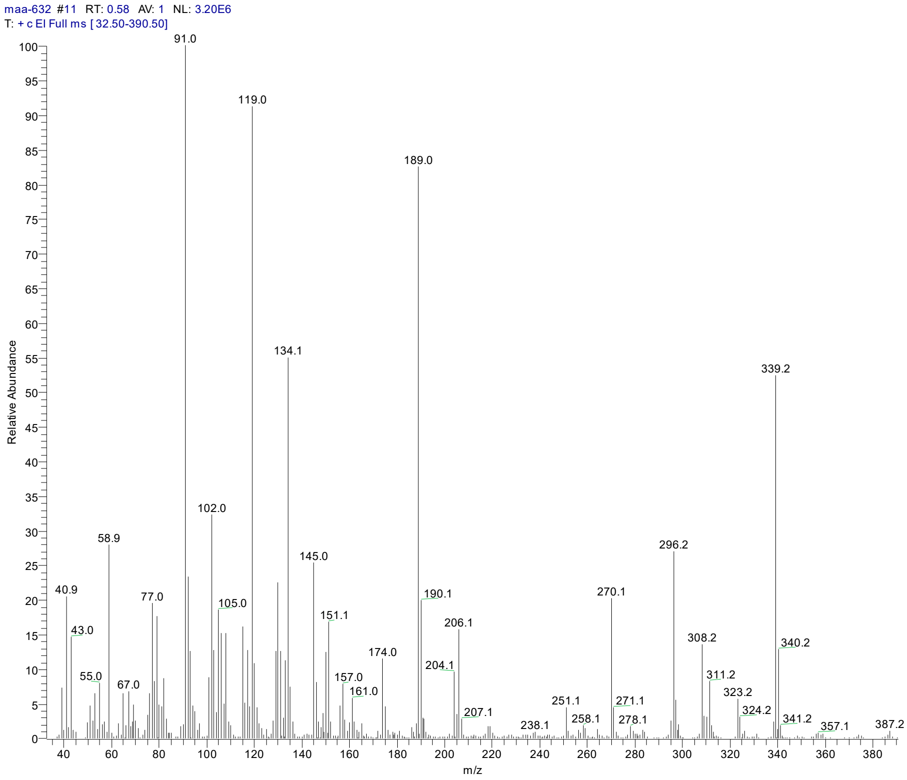

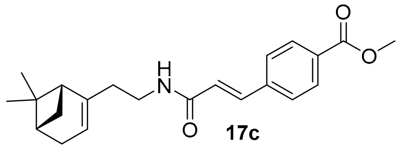

17c

^{1}H NMR spectrum of compound **17c**

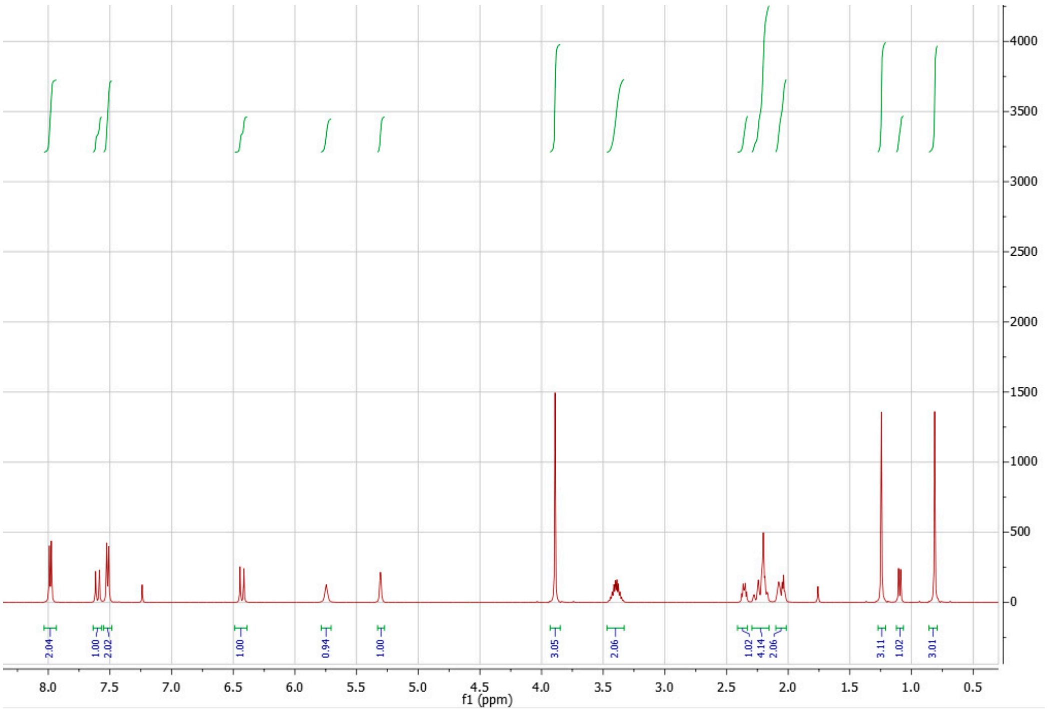

^{13}C NMR spectrum of compound **17c**

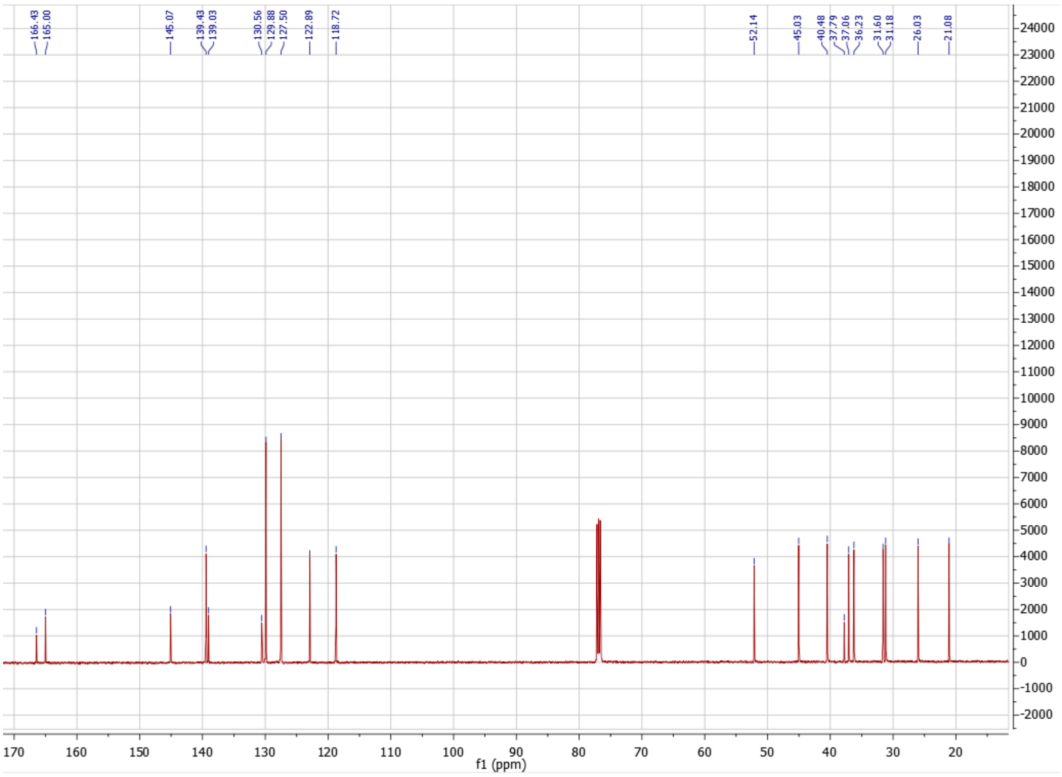

Mass-spectrum of compound **17c**

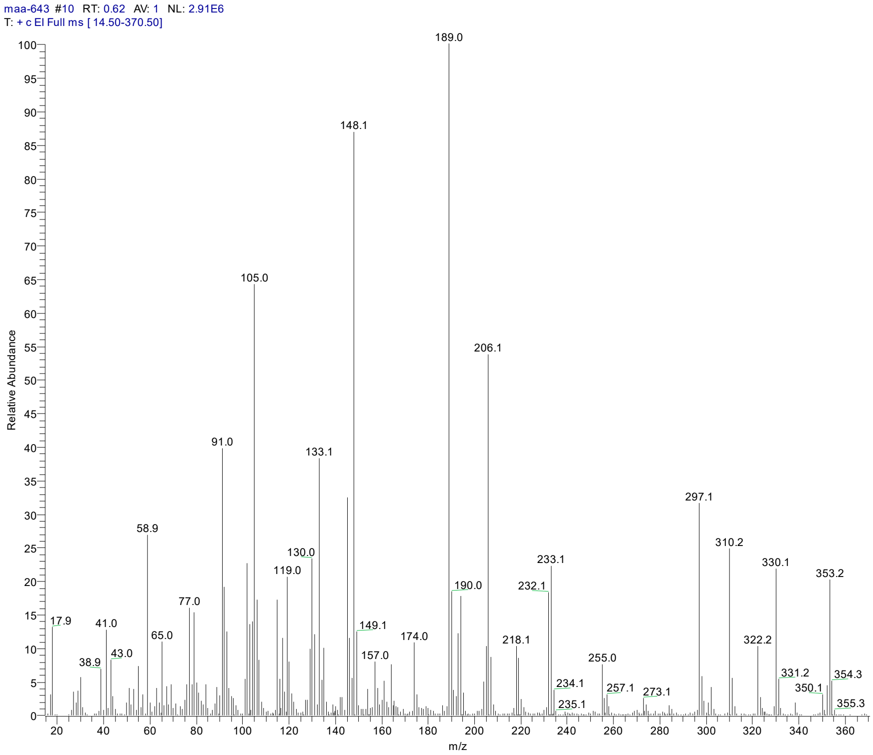

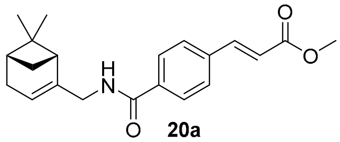

20a

¹H NMR spectrum of compound **20a**

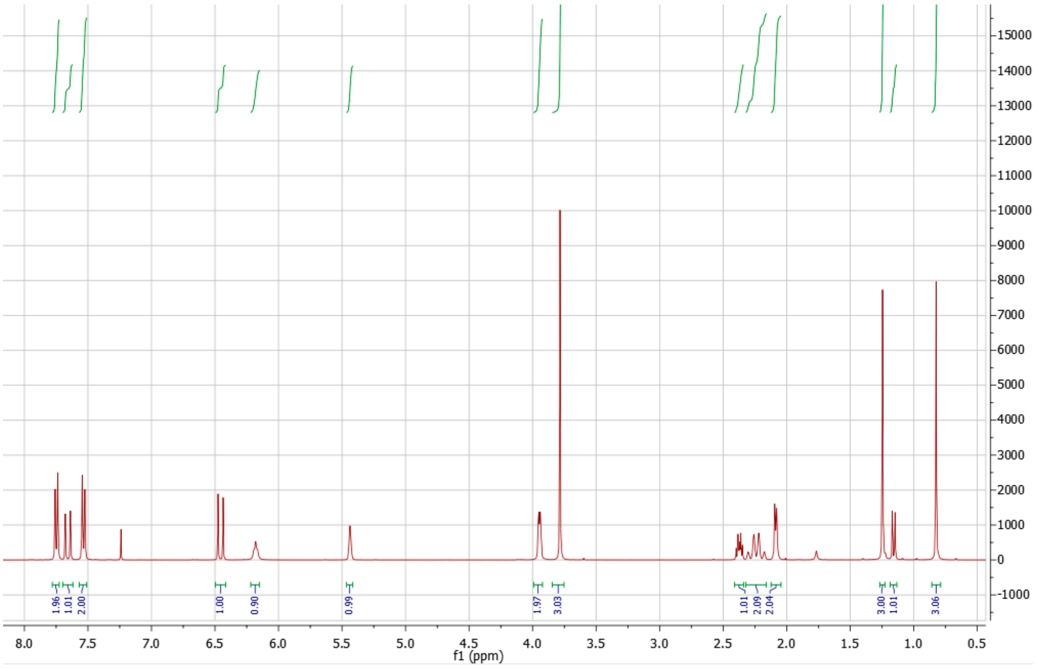

¹³C NMR spectrum of compound **20a**

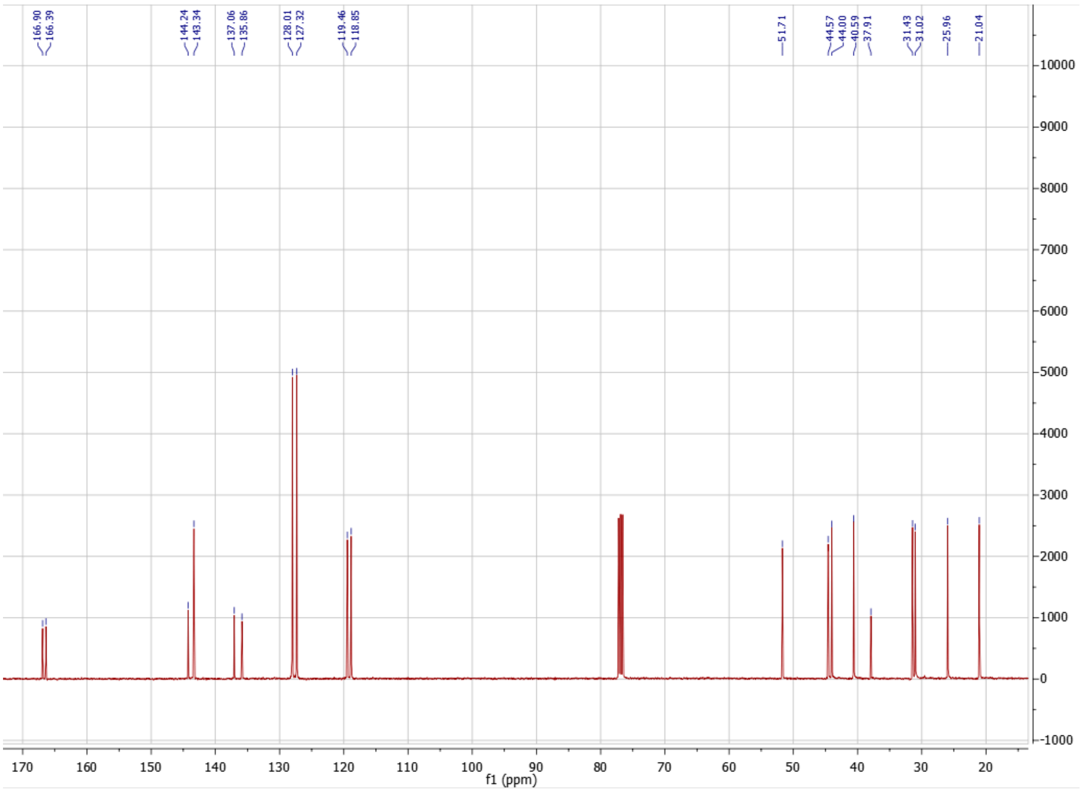

Mass-spectrum of compound **20a**

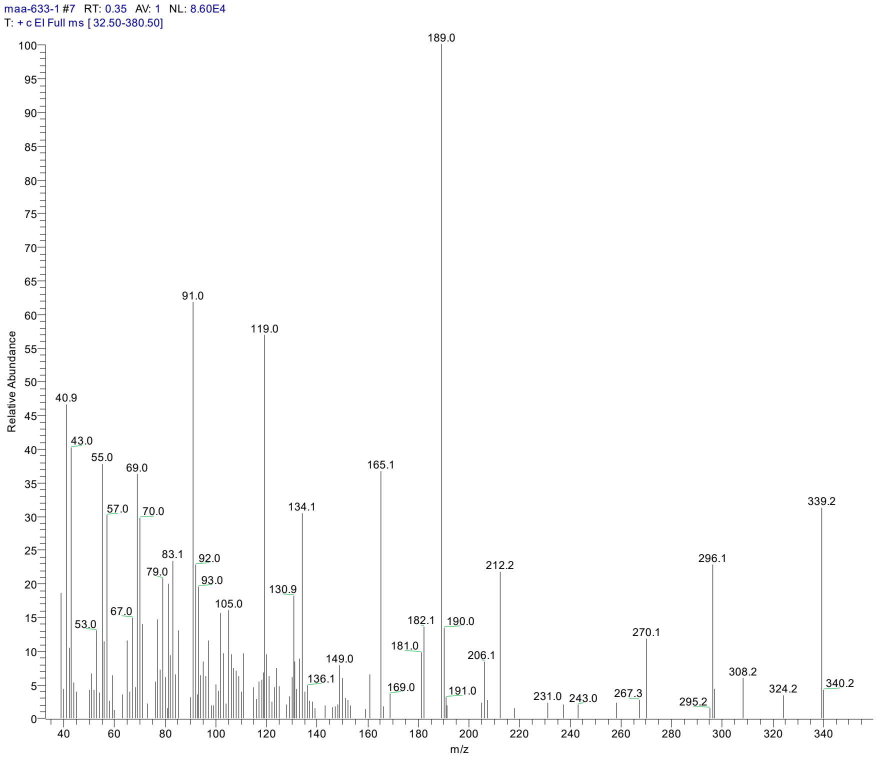

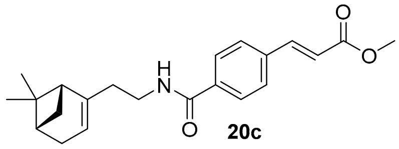

20c

^{1}H NMR spectrum of compound **20c**

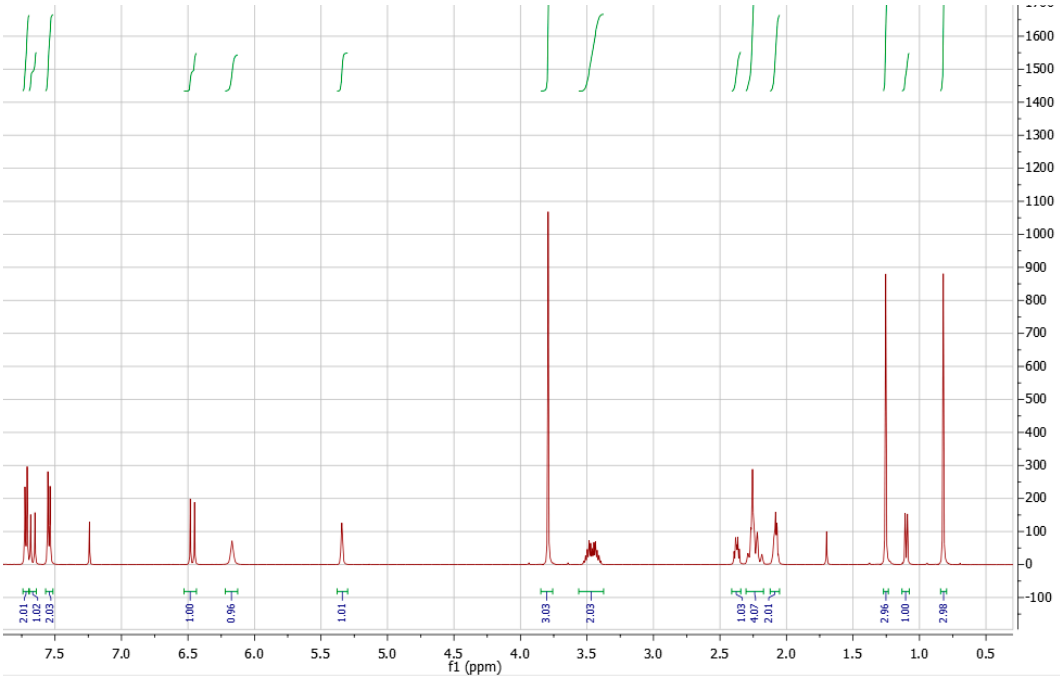

^{13}C NMR spectrum of compound **20c**

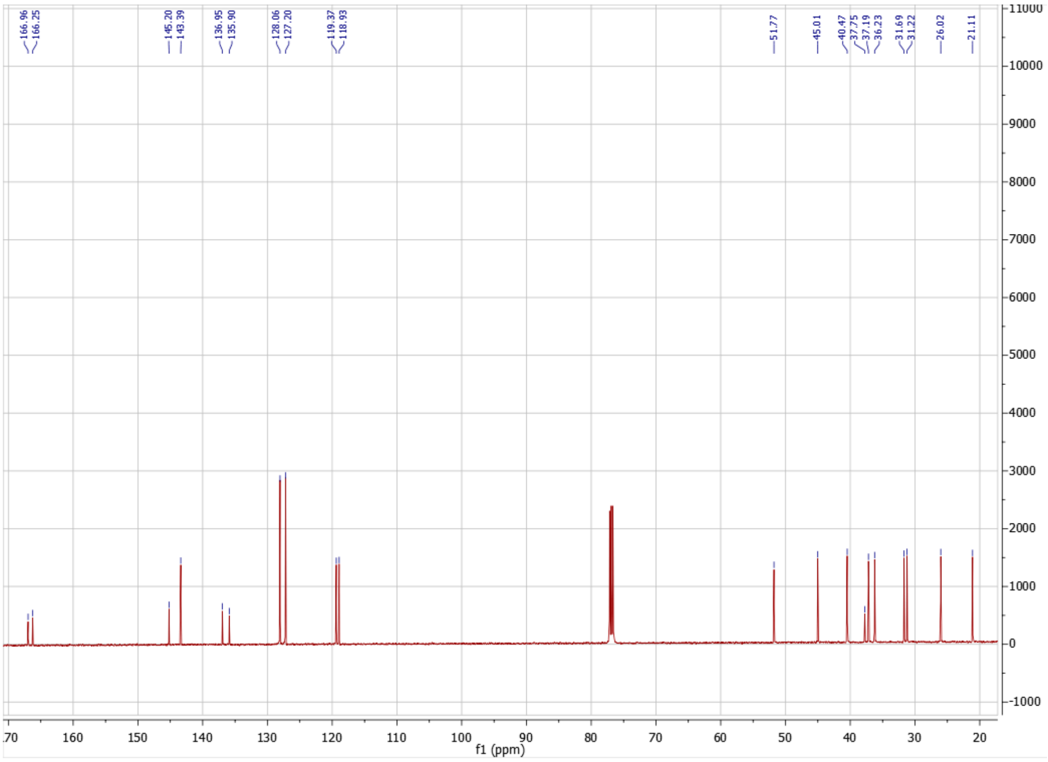

Mass-spectrum of compound **20c**

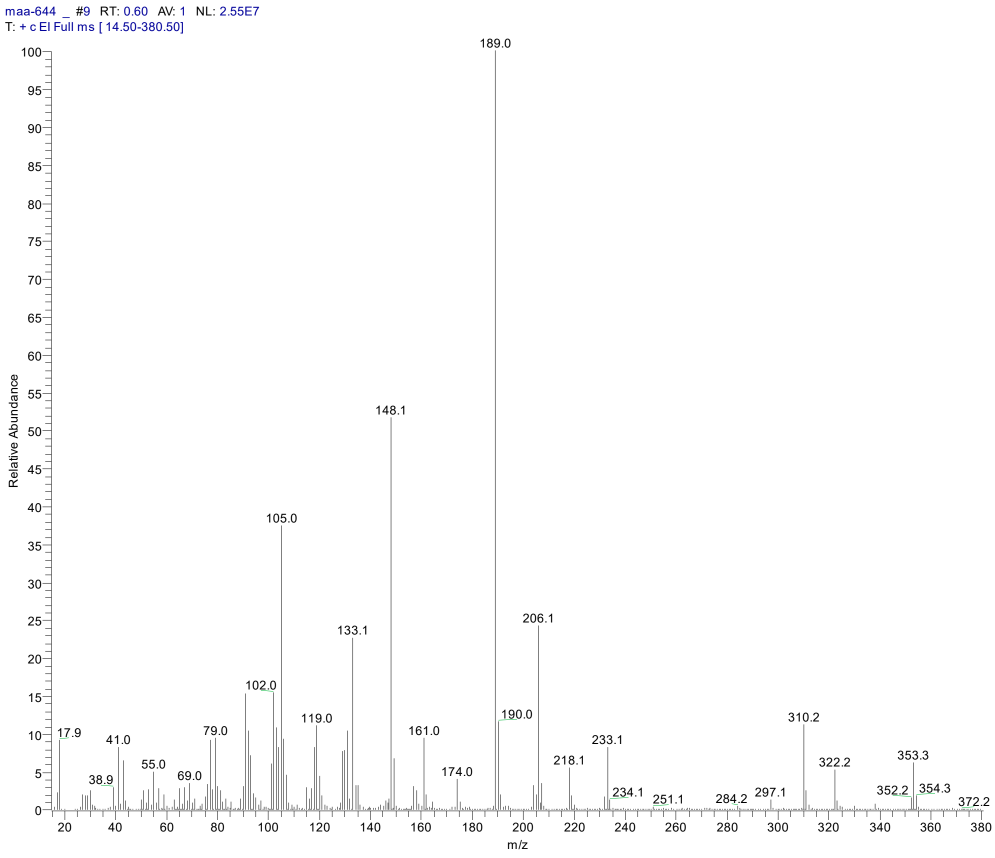

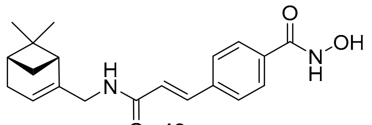

18a

^{1}H NMR spectrum of compound **18a**

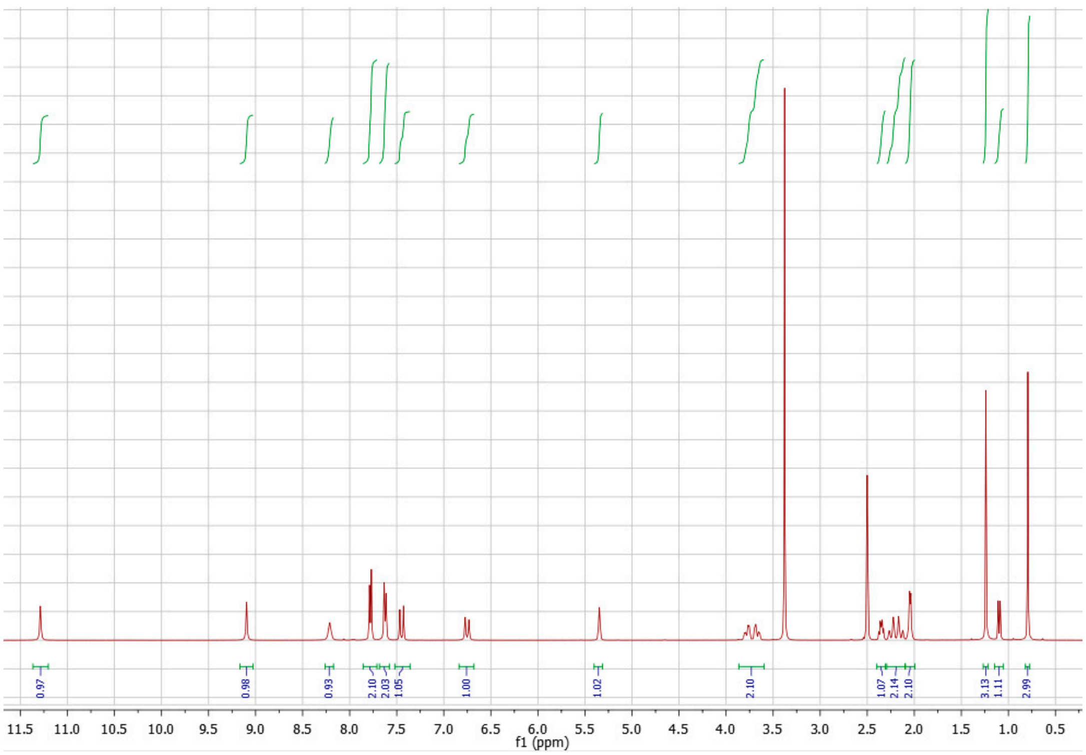

^{13}C NMR spectrum of compound **18a**

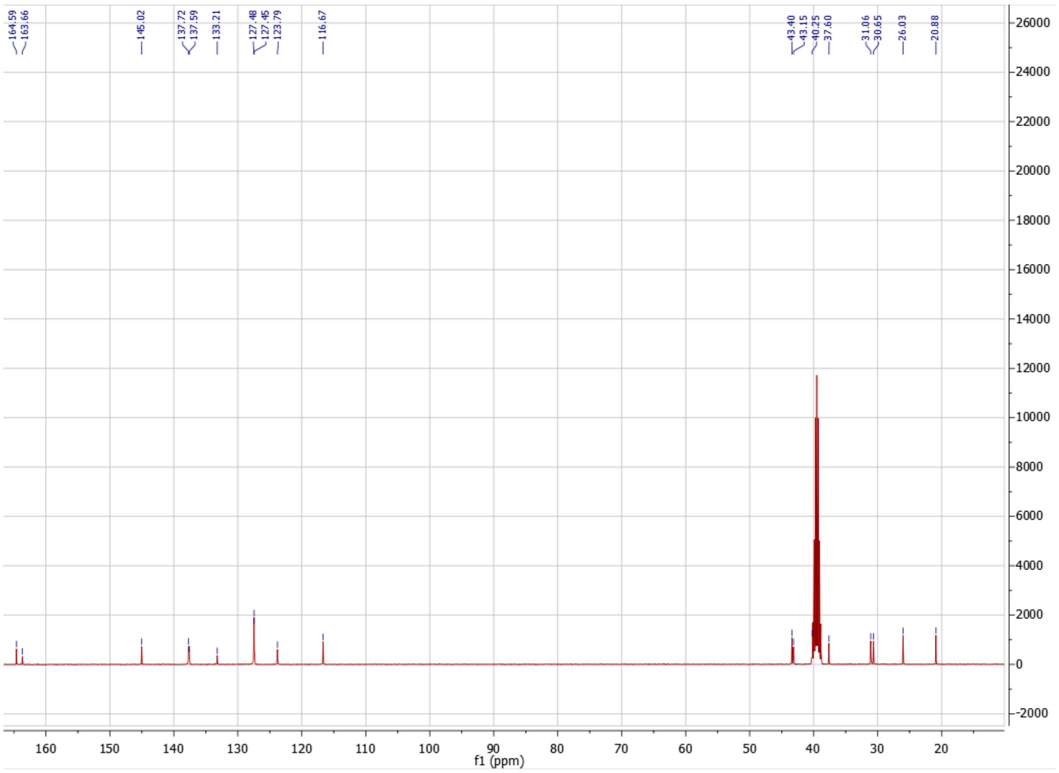

Mass-spectrum of compound **18a**

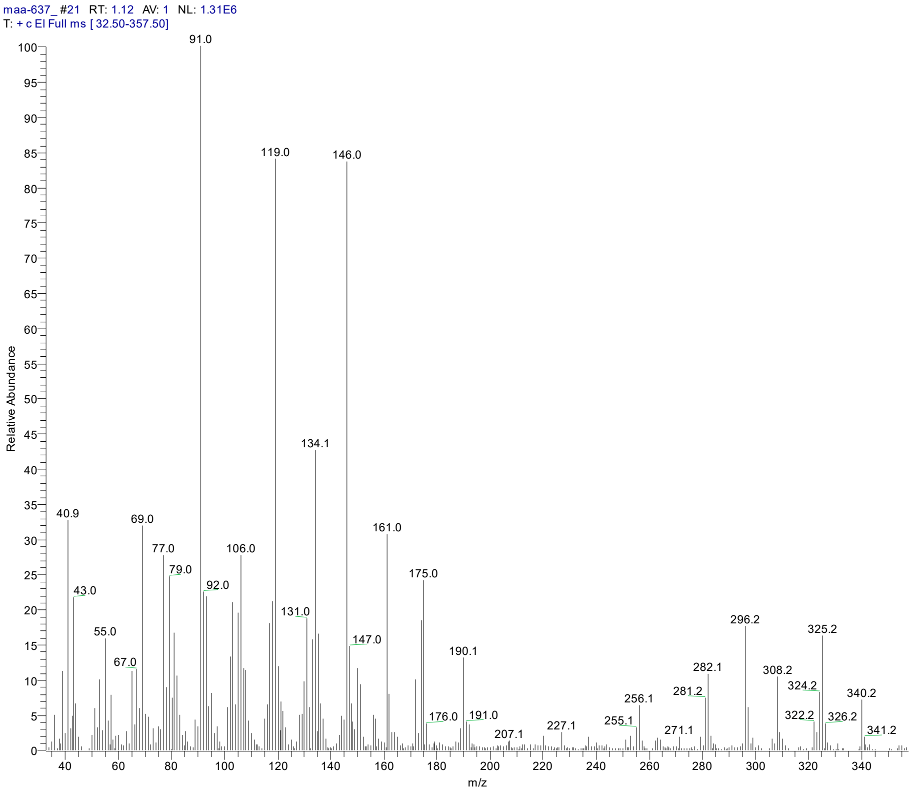

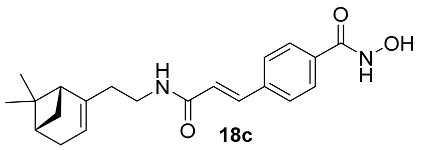

^{1}H NMR spectrum of compound **18c**

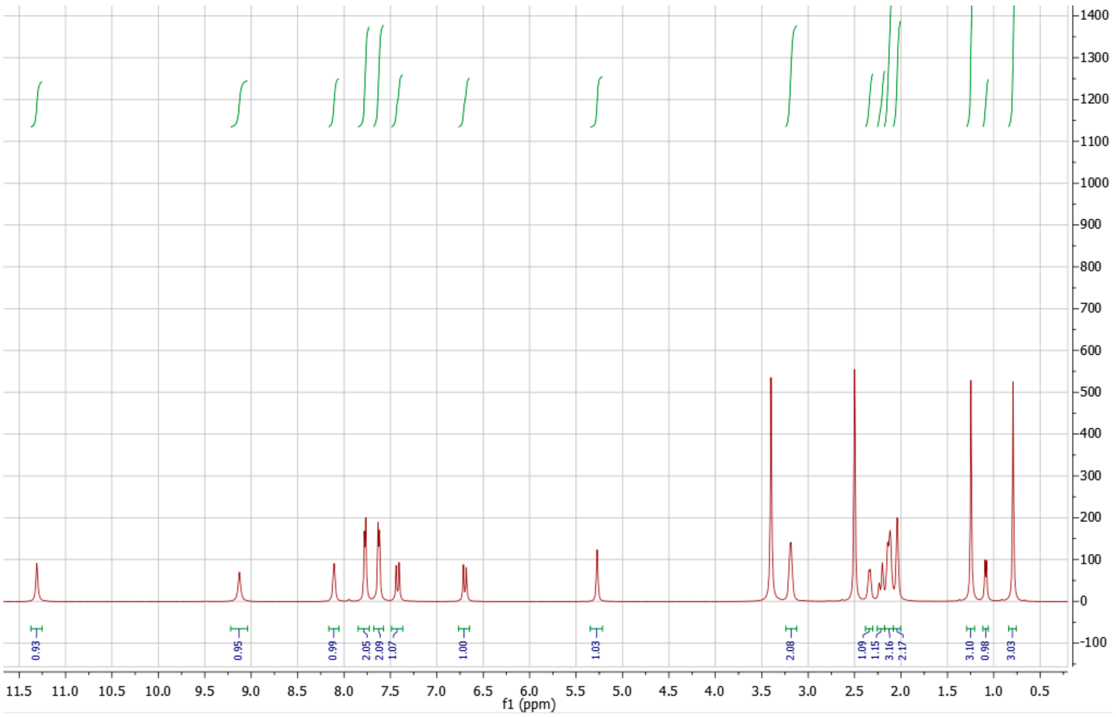

^{13}C NMR spectrum of compound **18c**

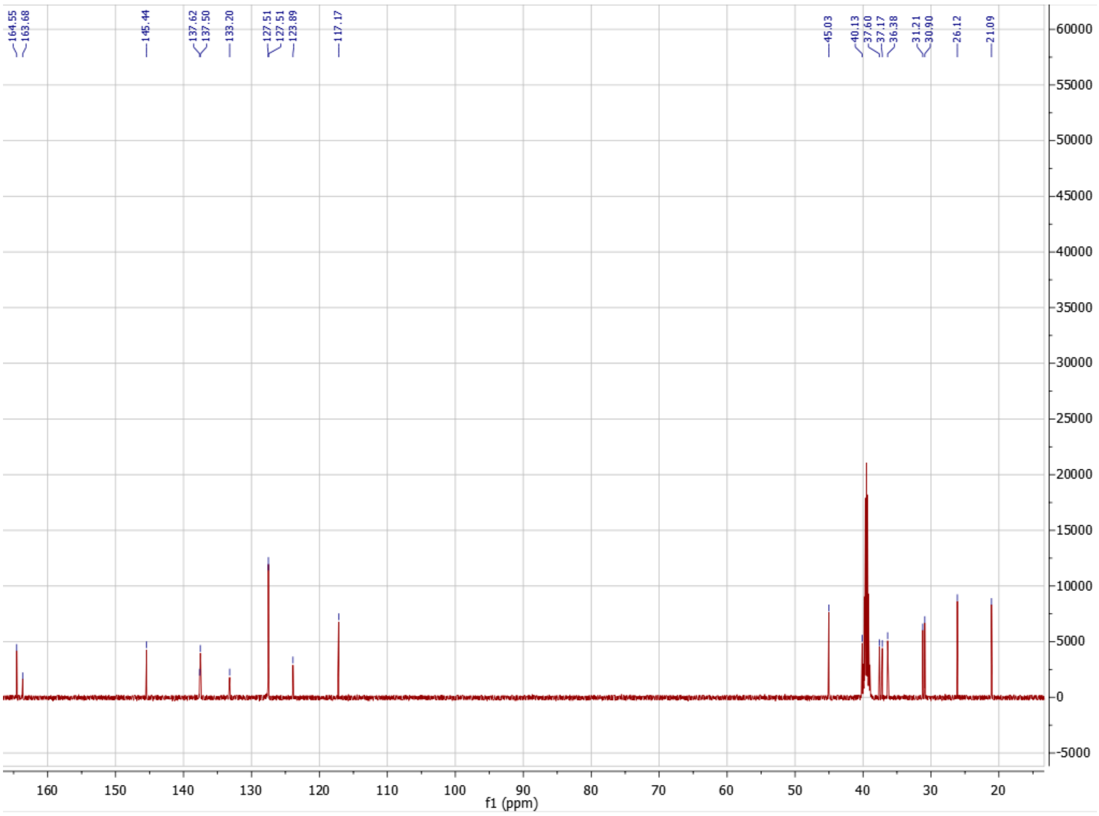

Mass-spectrum of compound **18c**

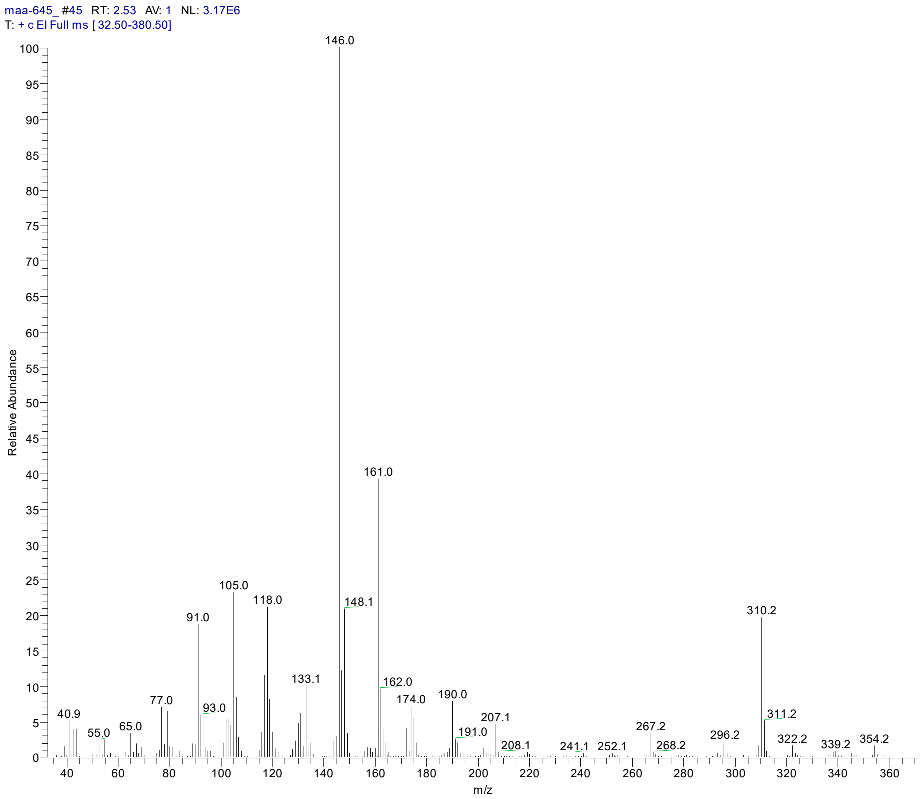

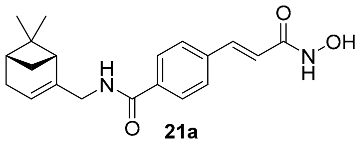

^{1}H NMR spectrum of compound **21a**

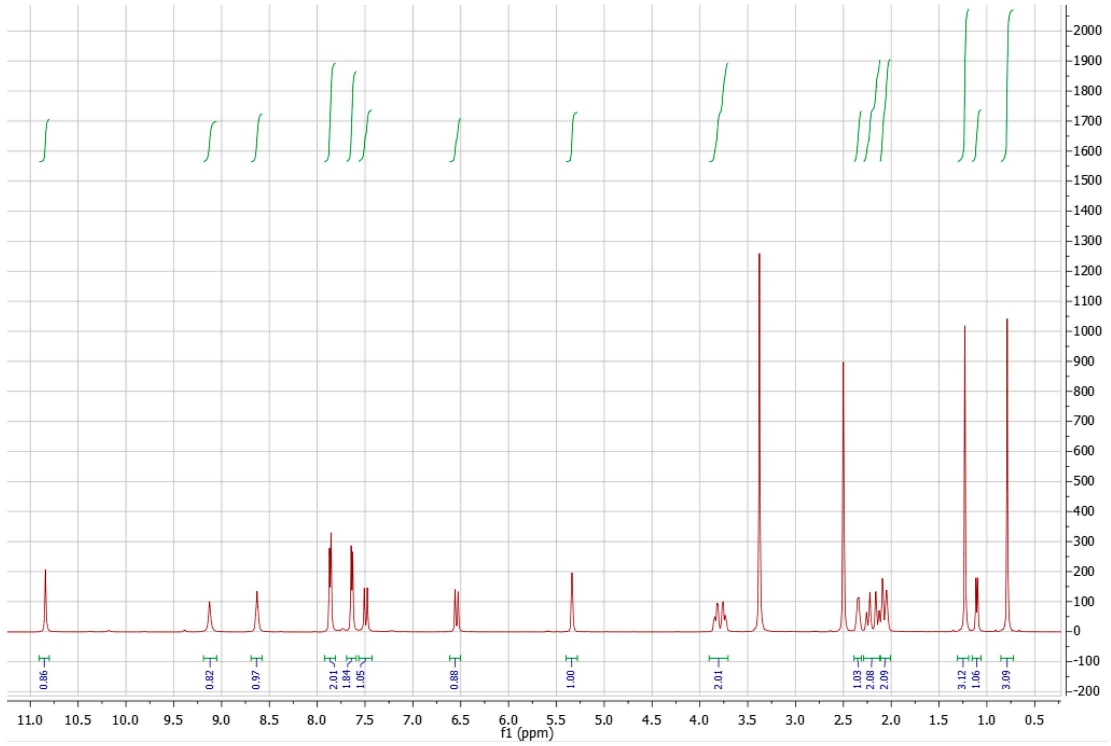

^{13}C NMR spectrum of compound **21a**

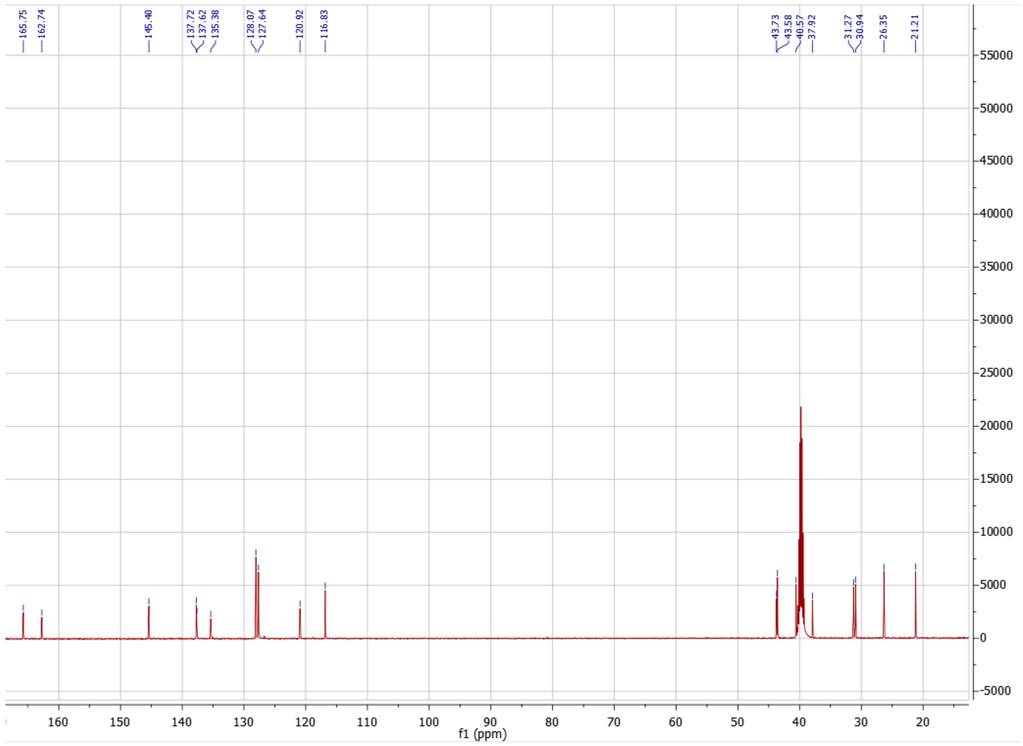

Mass-spectrum of compound **21a**

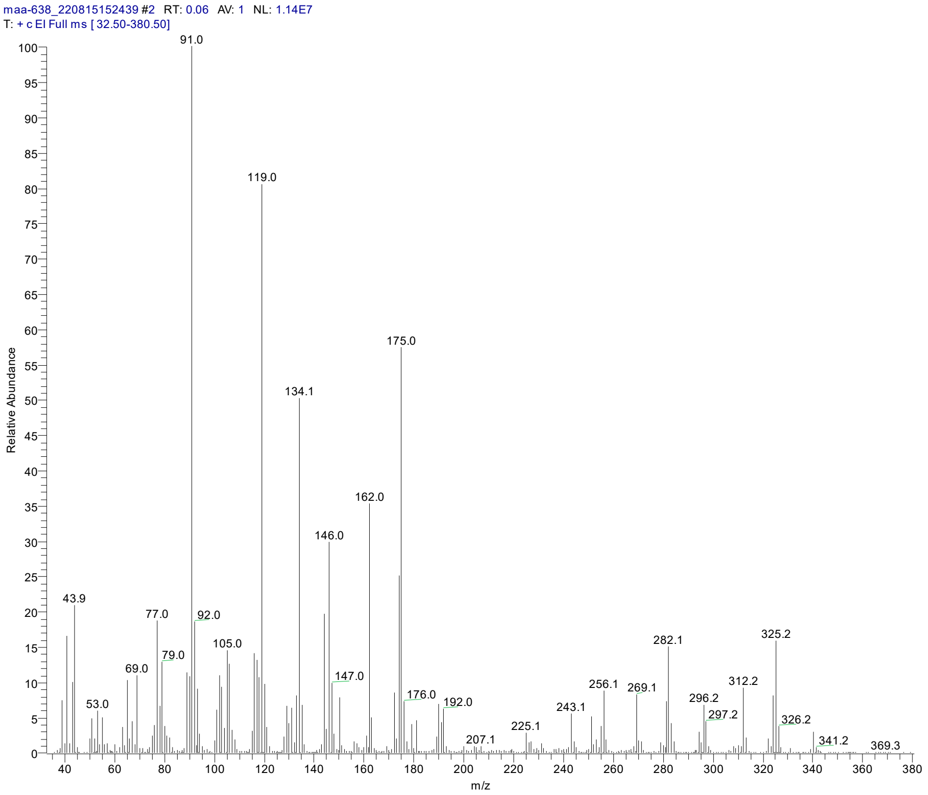

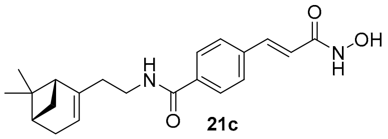

21c

^{1}H NMR spectrum of compound **21c**

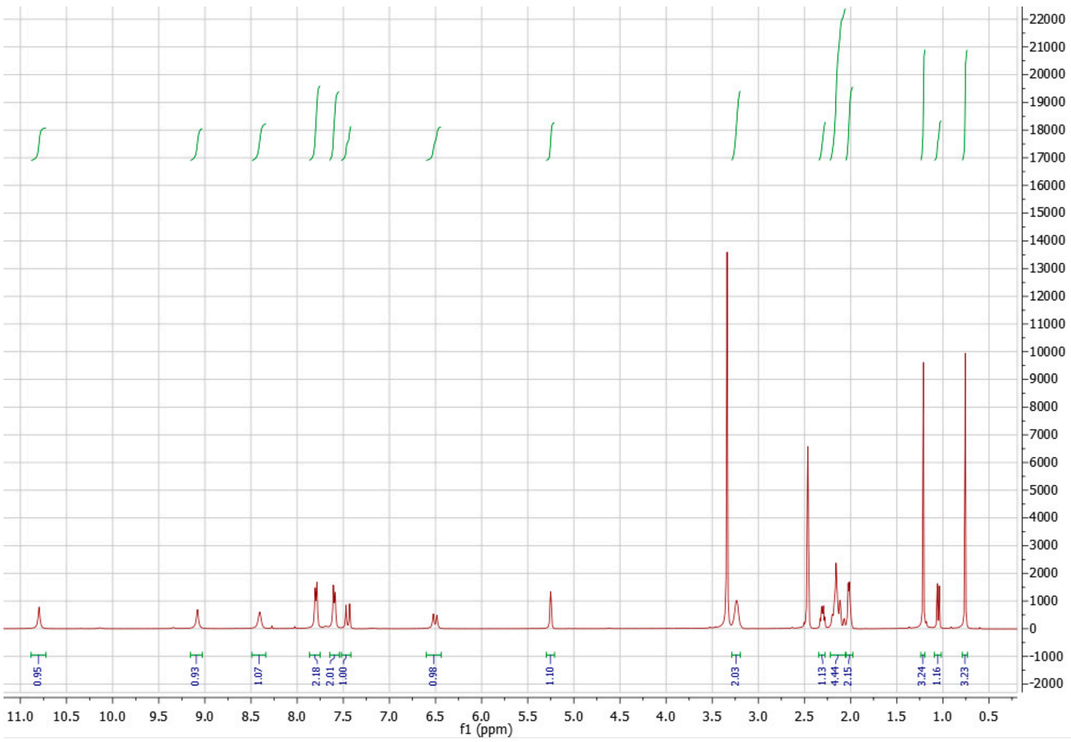

^{13}C NMR spectrum of compound **21c**

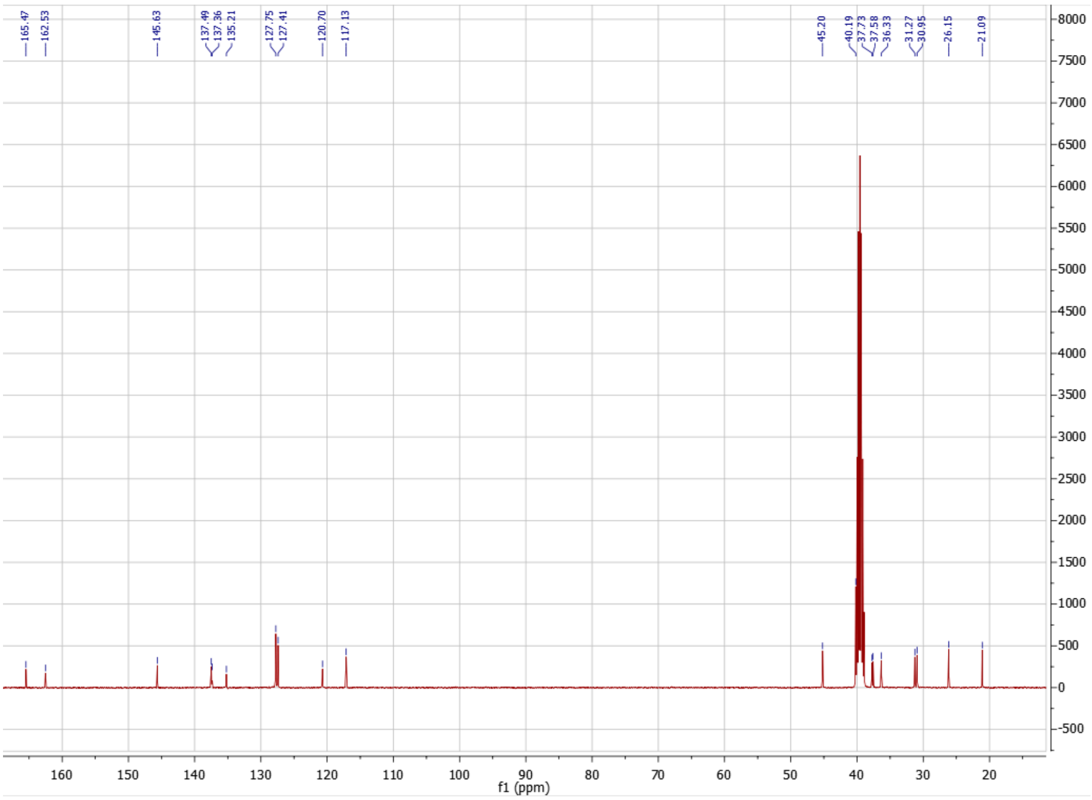

Mass-spectrum of compound **21c**

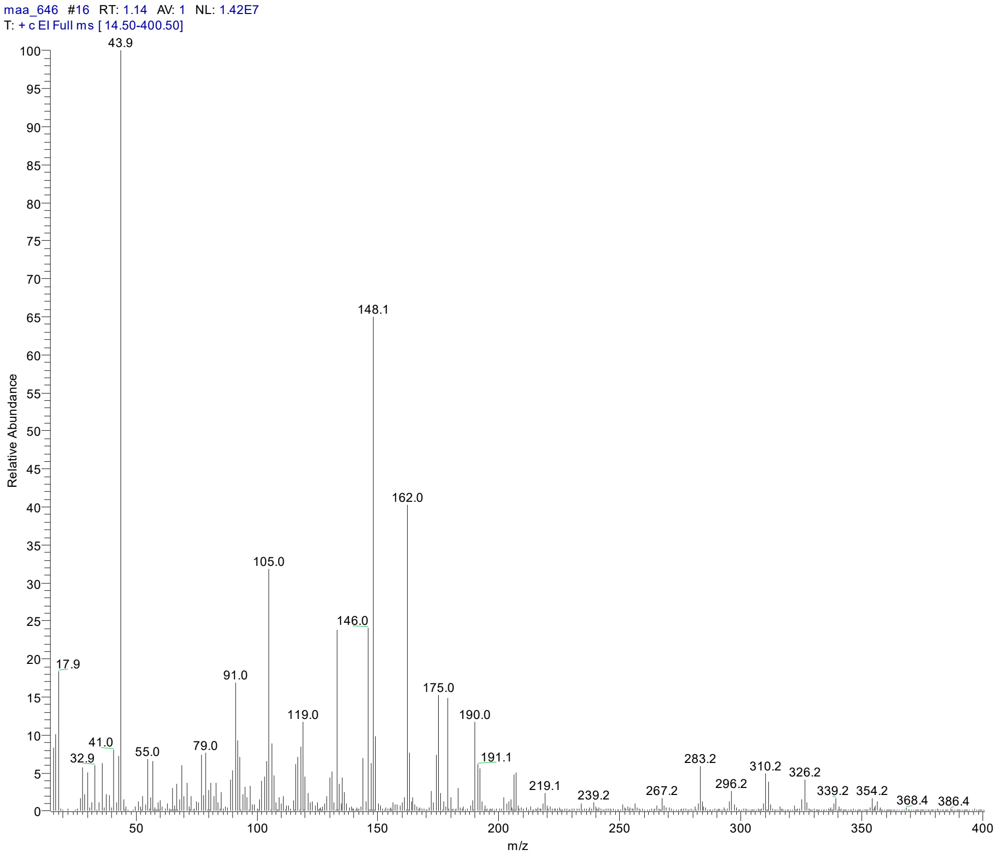

Figure S1. Molecular docking of ligands against Hexokinase 1 (PDB ID: 4F9O). Nitrogen and oxygen atoms are coloured in blue and red, respectively. Hydrogen bond (bright green dotted lines represents), van der Waals bond (light green dotted lines represents), other interactions (Alkyl are shown in light blue colour dotted lines; π-Alkyl - in light pink colour dotted lines; π-Anion - yellow colour dotted lines) Attractive charge are shown in beige colour dotted lines).

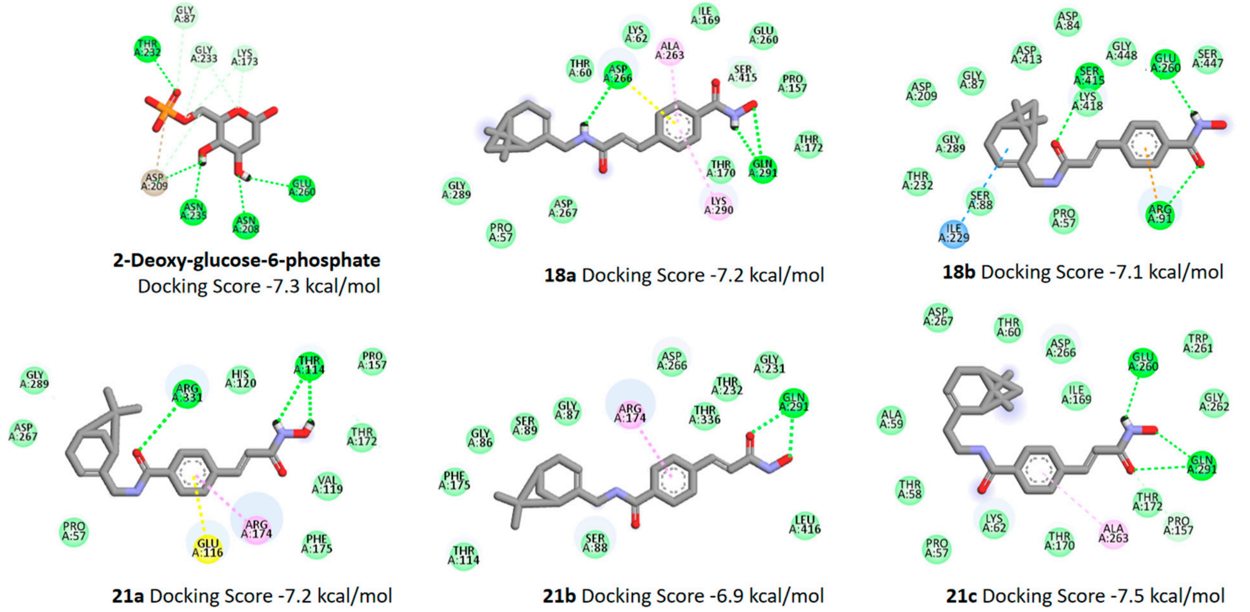

Figure S2. Molecular docking of ligands against 6-Phosphofructo-2-kinase (PDB ID: 1K6M). Nitrogen and oxygen atoms are coloured in blue and red, respectively. Hydrogen bond (bright green dotted lines represents), van der Waals bond (light green dotted lines represents), other interactions (Alkyl are shown in light blue colour dotted lines; π-Alkyl - in light pink colour dotted lines; π-π Stacked - in bright pink colour dotted lines, π-Cation - orange colour dotted lines).

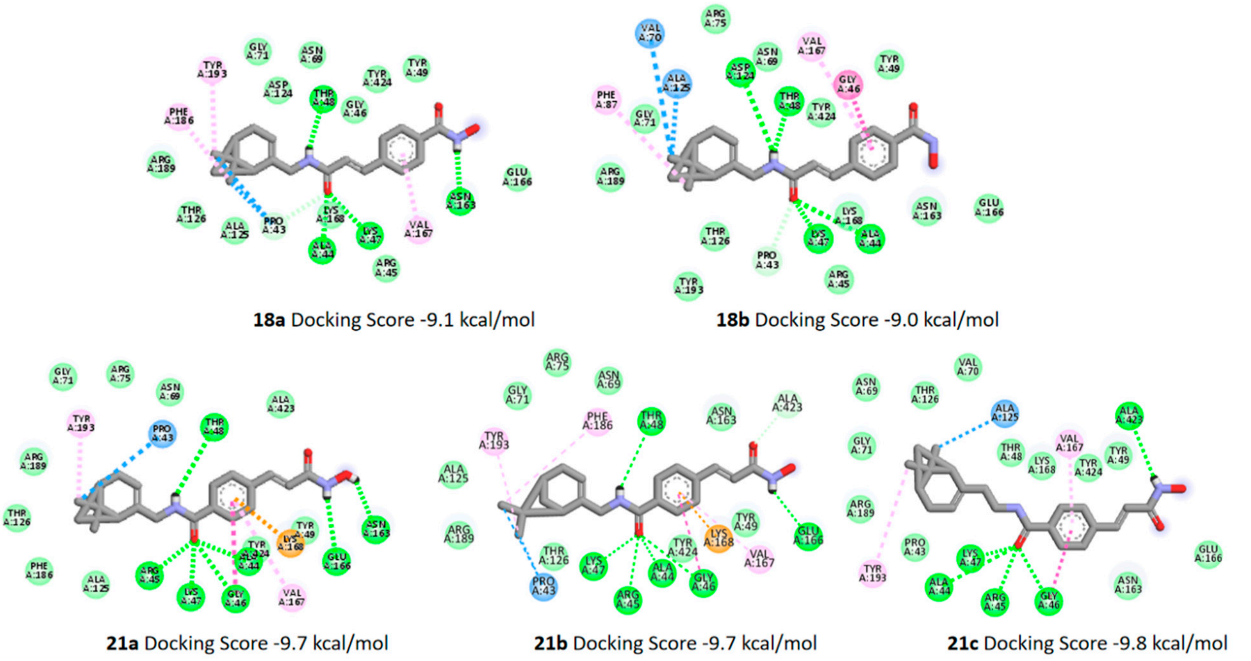

Figure S3. Molecular docking of ligands against Pyruvate kinase M2 (PDB ID: 1T5A). Nitrogen and oxygen atoms are coloured in blue and red, respectively. Hydrogen bond (bright green dotted lines represents),

van der Waals bond (light green dotted lines represents), other interactions (π-Alkyl - in light pink colour dotted lines; π-Anion and π-Cation - yellow and orange colour dotted lines, respectively).

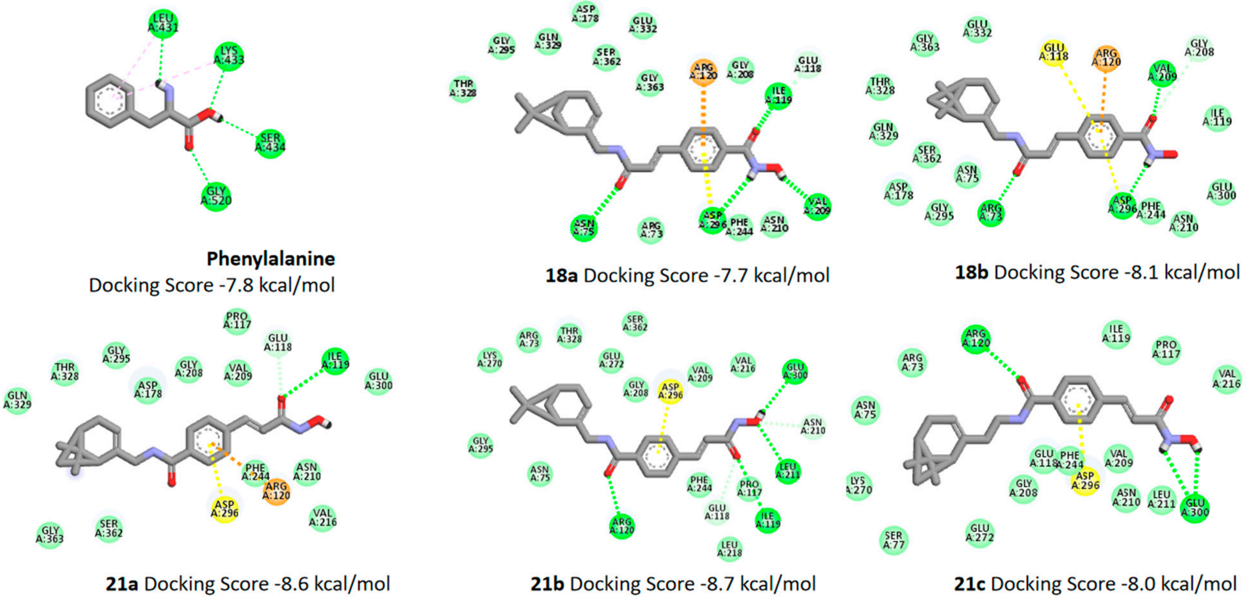

Supplement: Supplementary file 1 [file cancers-15-04985-s001.zip › cancers-2612337-supplementary.pdf]
